# Quantitative susceptibility mapping (QSM): Decoding MRI data for a tissue magnetic biomarker

**DOI:** 10.1002/mrm.25358

**Published:** 2014-07-17

**Authors:** Yi Wang, Tian Liu

**Affiliations:** 1 Radiology, Weill Medical College of Cornell UniversityNew York, New York, USA; 2 Biomedical Engineering, Cornell UniversityIthaca, New York, USA; 3 Biomedical Engineering, Kyung Hee UniversitySeoul, South Korea; 4 MedImageMetric, LLCNew York, New York, USA

**Keywords:** QSM, quantitative susceptibility mapping, gradient echo, metabolism, iron, oxygen consumption, ferritin, hemoglobin, hemorrhage, calcification, myelin, contrast agent, quantification, dipole field, dipole kernel, morphology enabled dipole inversion, Bayesian

## Abstract

In MRI, the main magnetic field polarizes the electron cloud of a molecule, generating a chemical shift for observer protons within the molecule and a magnetic susceptibility inhomogeneity field for observer protons outside the molecule. The number of water protons surrounding a molecule for detecting its magnetic susceptibility is vastly greater than the number of protons within the molecule for detecting its chemical shift. However, the study of tissue magnetic susceptibility has been hindered by poor molecular specificities of hitherto used methods based on MRI signal phase and T2* contrast, which depend convolutedly on surrounding susceptibility sources. Deconvolution of the MRI signal phase can determine tissue susceptibility but is challenged by the lack of MRI signal in the background and by the zeroes in the dipole kernel. Recently, physically meaningful regularizations, including the Bayesian approach, have been developed to enable accurate quantitative susceptibility mapping (QSM) for studying iron distribution, metabolic oxygen consumption, blood degradation, calcification, demyelination, and other pathophysiological susceptibility changes, as well as contrast agent biodistribution in MRI. This paper attempts to summarize the basic physical concepts and essential algorithmic steps in QSM, to describe clinical and technical issues under active development, and to provide references, codes, and testing data for readers interested in QSM. Magn Reson Med 73:82–101, 2015. © 2014 The Authors. Magnetic Resonance in Medicine Published by Wiley Periodicals, Inc. on behalf of International Society of Medicine in Resonance. This is an open access article under the terms of the Creative commons Attribution License, which permits use, distribution, and reproduction in any medium, provided the original work is properly cited.

## Introduction

Magnetic susceptibility is one of the following major categories of tissue contrast mechanisms in proton MRI 1: 1) spin thermal relaxation in a voxel of water; 2) water motion, including diffusion, perfusion, flow and tissue deformation; and 3) molecular electron cloud polarization by the main magnetic field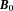. A polarized molecule generates its own magnetic field, which is known as a chemical-shift shielding field for observer protons inside the molecule and as a magnetic-susceptibility inhomogeneity field for observer protons outside the molecule. This field adds phase accumulation and consequently causes intravoxel dephasing or magnitude T2* decay in the commonly available gradient echo (GRE) MRI. Therefore, noninvasive MRI is well suited for investigating the magnetic susceptibility of tissue. The GRE phase is equal to the magnetic field multiplied by the gyromagnetic ratio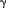 and the echo time (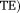. This phase may be used to further attenuate the signal for enhancing T2* image contrast, which is called susceptibility weighted imaging 2–4. However, the field at an observer location is the sum of contributions from all surrounding magnetic susceptibility sources, with each contribution dependent on the source-observer distance and orientation 5. Consequently, the phase or T2* contrast does not exclusively depict the local tissue magnetic property but is a weighted summation of the magnetic properties of the surrounding tissue, reflecting only the “shadow” of the surrounding susceptibility sources. For example, the phase and T2* contrast of tissues with weak susceptibility may primarily come from nearby air-tissue interfaces, across which there are large susceptibility changes. To uncover local tissue magnetic properties, the field has to be deconvolved, which is referred to as quantitative susceptibility mapping (QSM).

QSM was contemplated at the early days of MRI 6. However, the inversion from field to susceptibility is ill-posed 7,8: There are zeroes in the kernel connecting the susceptibility distribution and the field, and a simple kernel division causes large errors that present as streaking artifacts in the reconstructed susceptibility map 9,10. Regularization or conditioning is necessary to select a unique solution for a given field 10–16. Fortunately, MRI provides plenty of information on tissue anatomical structures. This information can serve as a prior in Bayesian regularization to overcome this ill-posed inverse problem, generating a reasonably accurate susceptibility map 17–20. Various regularizations have since been developed 17,19–30, making QSM a feasible tool for the MRI community.

**Figure 1:**
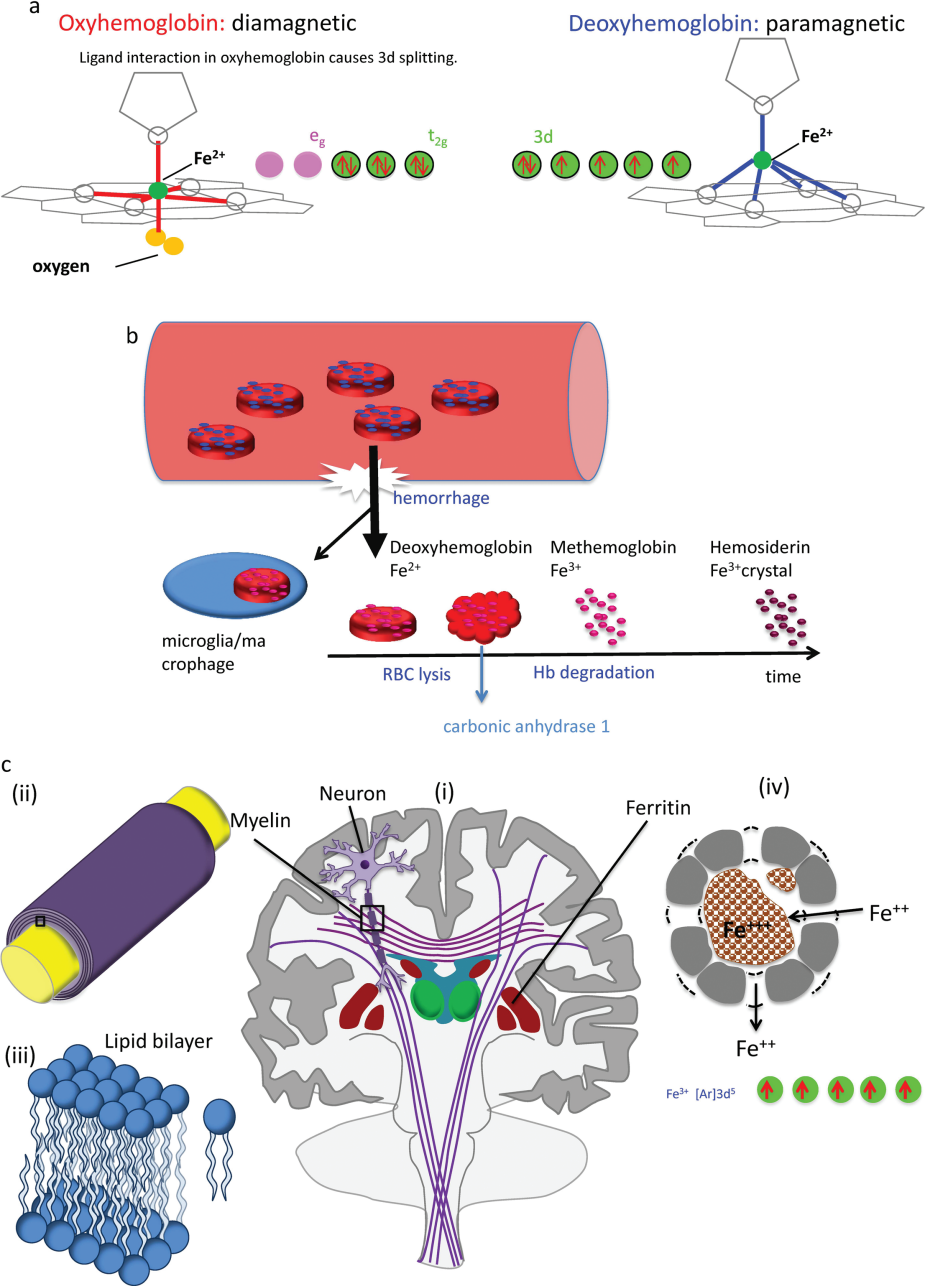
Biomedical magnetic materials. (a) Diamagnetic hemoglobin and paramagnetic deoxyhemoglobin. During metabolic consumption of oxygen in the brain, heart, and kidney, weakly diamagnetic oxyhemoglobin releases O_2_ and becomes strongly paramagnetic deoxyhemoglobin. Whereas the 3d electron orbits of Fe^2+^ in deoxyhemoglobin may be approximated as an isolated iron atom with four unpaired electrons (right), the intramolecular interaction between the porphyrin ring and Fe^2+^ in oxyhemoglobin (ligand interaction) splits the Fe atom’s 3d-orbit into two levels, e_g_ and t_2g_, with all six electrons paired in the three t_2g_ orbits. (b) Blood degradation in hemorrhage. Following the onset of a hemorrhage, a small fraction of red blood cells (RBCs) may be endocytosed by microglia/macrophages. The majority of RBCs undergo cell lysis and hemoglobin (Hb) degradation from deoxyhemoglobin into methemoglobin (Fe^3+^) and hemosiderin (possible magnetic domain). Modeled after: Lancet Neurol 2012;11:720–731. (c) Susceptibility sources in the human brain. Major susceptibility sources in (i) the brain include myelin and ferritin. The white matter tracts in the brain consist of myelinated nerve fibers. (ii) Zoomed view of the box in (i) showing axon (yellow) and myelin sheath (purple). Myelin consists of several layers of lipid bilayer. (iii) Zoomed view of the box in (ii) showing a lipid bilayer and an individual lipid. (iv) Ferritin in a cross-section. Ferritin consists of a peptide spherical shell 2-nm thick with a 8-nm diameter cavity. Fe^2+^ enters through a four-fold symmetric channel, is stored as Fe^3+^ oxide mineral, and is released as Fe^2+^ through a three-fold symmetric channel. There are five unpaired 3d electrons in Fe^3+^, generating strong paramagnetism.

As indicated by a PubMed search of QSM papers (6 in 2011; 18 in 2012; and 37 in 2013), there is rapidly growing interest in developing techniques for QSM data acquisition and processing, and in developing clinical and scientific applications ranging from iron distribution and metabolic consumption of oxygen to myelin in white matter (WM) tracts (Fig. 1). This review tries to serve these interests by summarizing the basic physical concepts in QSM, outlining the fundamental algorithmic steps in QSM, organizing the available MATLAB (MathWorks, Natick, MA) codes for QSM algorithms, and surveying the clinical and technical QSM issues that are under active development.

### Preparation: Estimating Susceptibility-Generated Field from its Effects on MRI Signal

In the MRI main field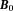, a molecule in tissue gains a magnetic moment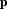 through its electron cloud polarization. Correspondingly, a tissue with volume magnetic susceptibility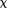 gains magnetization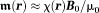 (see TISSUE MAGNETISM in supporting information for a brief summary of the molecular physics). Tissue magnetization generates its own magnetic field that affects MRI signal. Here we review the mathematical relationships that link magnetization, field, and MRI signal, based on which the field can be estimated from the MRI signal.

### Magnetic Dipole Field and Field Observed by a Proton in Tissue

According to Maxwell’s equations in vacuum, a magnetic dipole moment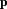 at a source location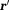 generates a magnetic field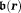 at an observation location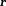 5 (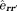 is a unit vector along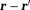),

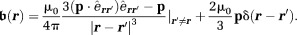
1

**Figure 2:**
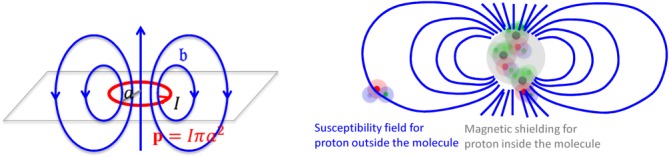
Magnetic fields, chemical shift, and magnetic susceptibility.(Left) The field of a magnetic dipole modeled by a current loop of radius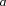. At the loop center,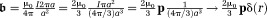 as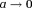.(Right) The electron cloud of a molecule polarized by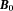 generates magnetic shielding or chemical shift for the observer proton in the molecule and a susceptibility field (in dipole pattern) for the observer proton outside the molecule.

Here, the inverse-cube of the distance term characterizes the spatial extent of the dipole field. The delta-function term can be understood from the field of a current loop with a fixed magnetic moment and a radius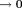 (Fig. 2). In water MRI, the delta-function term is dropped; the probability of the polarized electron cloud penetrating into the space of the observer water protons is negligible. Thus, the field (scaled to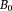) observed by a water proton is the sum of contributions from all surrounding susceptibility sources [their distribution defined by magnetization
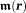
**]**, excluding that from the proton’s own location:

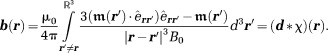
2

The exclusion of the observer point in the integration of Eq. 2 represents the Cauchy principal value, commonly known as the Lorentz correction. This is conventionally, but unnecessarily, interpreted as subtracting a sphere of magnetization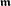 that has a uniform interior field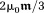 from the sum of the fields of all sources according to Eq. 1 5. In Eq. 2,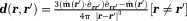. (Here [expression] – 1 if expression is true and 0 otherwise.)

Eq. 2 relates the field at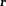 to the magnetization distribution over the whole space. This can be expressed in a differential form that relates the field at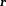 to the magnetization located at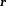 31:

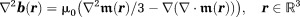
3

Eq. 3 can be Fourier transformed into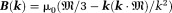. For notational convenience, we use lower case for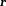-space quantities and upper case for corresponding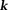-space quantities (except constant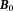), the z-component (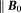) of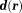 is referred to as the dipole kernel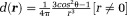 with Fourier transform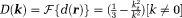, and the z-component of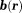 is noted as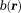. For scalar susceptibility, Eq. 2 becomes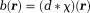 with Fourier transform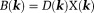.

On the other hand, the electron cloud of a molecule does penetrate into and interact with observer protons within the molecule. Consequently, electron cloud polarization by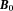 induces a shielding magnetic field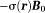 (Fig. 2) 32, or chemical shift (referenced to water), that alters the field experienced by protons within the molecule: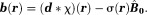 Both the magnetic susceptibility (observed by a large number of water protons outside the molecule) and the chemical shift (observed by protons inside the molecule) reflect the same molecular electron-cloud polarization 33–35.

### Field Effects on MRI Signal

The magnetic field of a polarized molecule may affect the MRI signal magnitude through a chemical exchange between free water and water bound to the molecule (inner sphere relaxation) and through a free water diffusion in the field (outer sphere relaxation) 36. These complicated effects are characterized as relaxation enhancement 37–41. Susceptibility estimation from MRI signal magnitude affected by relaxation is prone to large errors 31. Fortunately, the phase of a water proton spin linearly increases with the field. Using multiple radio frequency (RF) coils, with the
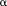
th coil element having a complex coil sensitivity function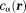 and acquisition noise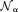, the ***k***-space signal measured in coil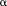 at time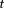 is 1:

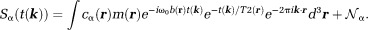
4

Here,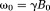 and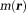 is the proton transverse magnetization [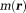
is proportional to proton density and is much smaller than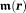 the electronic magnetization]. Typically,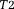 is homogeneous within a voxel and is much longer than the readout duration (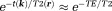), and the data sampling gradient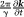 is much larger than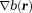 (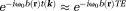). Then, the complex image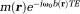 can be reconstructed using the Moore-Penrose pseudo-inverse 1 for both full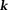 -space sampling 42 and parallel imaging 43. Approximating the voxel sensitivity function 44 as a box function, the signal detected at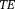 in a voxel centered at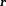 with size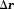 (volume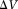) is the summation over signals from all proton spins in the voxel,

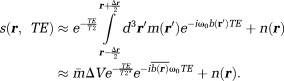
5

Here, the integration is evaluated by assuming small phase inhomogeneity in a voxel: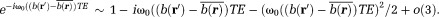 The quantities in Eq. 5 are defined as:
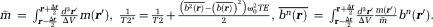

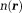
 is the noise in the reconstructed image. Common imaging situations involve weak tissue susceptibilities, short TEs (and small echo spacing in multiple echo sampling), and small voxels. Then, the phase of the signal in a voxel is approximately equal to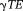 times the proton density-weighted average of the field in the voxel, and the T2* decay rate differs from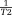 by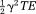 times the proton density-weighted field variance in a voxel. As a result, MRI phase signals allow us to measure the magnetic field generated by tissue susceptibility.

### Pulse Sequences Sensitizing Susceptibility

The three-dimensional (3D) GRE sequence is currently the most frequently used to acquire data for QSM. Multiple echoes (number of echoes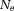 – 1–12 having been reported in literature) can be used with short TE for detecting strong susceptibility and long TEs for weak susceptibilities. The GRE sequence on a typical 3T MRI system may allow a high-resolution acquisition (∼0.5 × 0.5 × 2.0 mm^3^) with minimal first TE ∼5 msec, uniform TE spacing ∼5 msec, readout bandwidth ∼300 Hz/pixel, last TE ∼45 msec, and TR ∼50 msec (whole brain scan time ∼5–10 min). For imaging regions with very strong susceptibility variations, such as those containing the bone-tissue and air-tissue interfaces, ultra-short 45,46 or zero TE 47 and small TE increments may be used to achieve adequate signal-to-noise ratio (SNR). Flow compensation can be used to account for phases caused by motion 48. Resolution should be as high as possible to minimize averaging effects in Eq. 5. Parallel imaging and spiral sampling trajectories 49,50 can be used to reduce scan time. Susceptibility effects may also be detected with asymmetric spin echo (SE) sequences. It is also possible to measure the magnetic field in a voxel through direct saturation of the water protons at a range of off resonance frequencies and Lorentzian lineshape fitting (water saturation shift referencing) 51, similar to continuous-wave NMR spectroscopy. Whereas GRE sequences generally are faster than SE sequences, there may be applications that benefit from a combination of GRE and SE acquisitions.

### Digitized Dipole Field: Field and Susceptibility Averaged over a Voxel and Localized at the Voxel Center

The field averaged over a voxel in Eq. 5 requires digitization of Eq. 2. The assumption that the distributions of the observation protons and susceptibility sources are fairly uniform within a voxel (physical smoothness) may allow for digital interpretation of Eq. 2: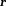 as the voxel center,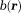 as the field value at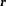, and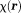 as the average of the susceptibilities in the source voxel positioned at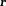. This digitization can be analytically derived for spherically shaped voxels: The field’s spherical mean value equals the field value at the sphere center 5; the field outside of a uniformly magnetized sphere is equivalent to the field of a dipole defined by the sphere’s total moment placed at the sphere’s center; and the field inside the sphere is zero (with the Lorentz correction by Eq. 2). The physical smoothness requirement is reasonably valid for high resolution MRI, and sinc-interpolation or zero-padding 44 may be used to reduce the digitization error. Accordingly, Eq. 2 will be used to model the MRI signal with voxels indexed by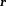.

### QSM Step 0: Estimate the Total Field from MRI Data

Eq. 5 models the MRI signal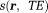 as a product of an exponential factor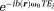 that describes a phase linear in time and a complex amplitude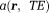 that contains a constant phase and an amplitude decay with time. Then, the total field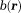 can be estimated from MRI signals at multiple TEs as a nonlinear least-squares problem per voxel 20,21,27:

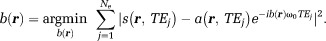
6

In the case of large SNR, Eq. 6 can be approximated as a linear regression of the signal phases at various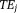, allowing for a closed form solution 21. In general, Eq. 6 can be solved iteratively and efficiently using a gradient search method, such as the Gauss-Newton 20 or Levenberg-Marquardt method 52, with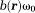 initialized as the linear rate of phase evolution between the first two echoes (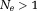). Eq. 6 may also be solved rapidly using autoregression 53. Interestingly, although Eq. 5 requires the estimation of complex coil sensitivity from calibration data 42,43, the field can be estimated without full knowledge of complex coil sensitivities from the phase difference at two TEs 54–57 or even at a single echo 58.

**Figure 3:**
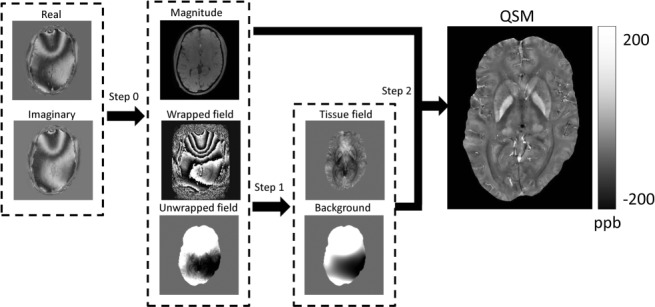
Schematics for quantitative susceptibility mapping. Quantitative susceptibility mapping (QSM) in general consists of three steps. Step 0: Generate magnitude image and field with unwrapping. Step 1: Remove background field and generate tissue field. Step 2: Generate QSM from tissue field and magnitude image.

The field estimated from Eq. 6 can contain many artificial jumps from voxel to voxel because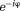 is periodic in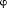 and can only define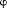 up to a period such as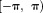. Consequently,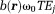 outside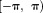 is aliased or wrapped into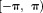, resulting in abrupt artificial jumps of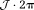 (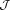 is an integer) in the output phase, which is called the wrapped phase denoted by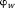. To estimate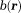, these phase wraps have to be compensated by adding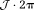 as needed:

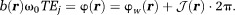
7


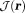
 is determined by the physical consideration that the unwrapped phase is spatially continuous. Unfortunately, it is difficult to impose continuity in discrete space because discretization is an undersampled approximation of the continuous space, and noise—ubiquitous in MRI data—causes jumps in regions of low SNR. There are many algorithms to impose continuity or smoothness in phase unwrapping 59, typically via path finding (including noncontinuous path, residual balancing, or branch cut) 60 or global error minimization (least squares) (Fig. 3). Phase can also be unwrapped rapidly by the Fourier spectral solution of the Laplacian of the phase 23,61; however, second order derivatives in the Laplacian may not allow large phase changes to occur in regions with big susceptibility variations, such as near veins 62. Phase unwrapping may be avoided for field data estimated from direct water saturation lineshape 51. A frequently used method is the path-finding type, image-quality-guided region growth 63, which works robustly and rapidly on MRI data using SNR as the image-quality guidance 17,64,65.

After phase unwrapping, a few bad points with fields not consistent with Eq. 2 may remain due to turbulent flow or idiopathic causes, and their effects can be reduced using a consistency check during QSM iteration 20. As the phase measurement in MRI is relative to RF carrier frequency, the field can only be determined up to a constant uniform field, which may be removed by background field removal in the next section.

### QSM Algorithm: Formulating and Solving the Field-to-Susceptibility Inverse Problem

The goal of QSM is to determine tissue susceptibility from the field (Eq. 6) or the complex MRI signal (Eq. 5) using Eq. 2, which connects the magnetic field and the tissue susceptibility. There are two fundamental challenges that are imbedded in Eq. 2 and Eq. 5, as outlined below, and we describe two QSM steps to address them correspondingly: 1) background field removal and 2) dipole inversion (Fig. 3).

### Two Fundamental Challenges in QSM

The first fundamental challenge is the lack of MRI signal in regions with susceptibility sources. MRI signal in Eq. 5 can only be detected in the region with water or the tissue of interest (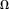). Magnetic susceptibility sources exist outside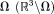, which is the background. However, there is no MRI signal in the background. If we regard each voxel in MRI as a field detector, then the number of detectors is less than the number of sources, making the field-to-source inverse problem ill-posed. Therefore, this lack of MRI signal in the background forms the first fundamental challenge for QSM. Susceptibility sources in tissue (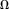) generate the tissue field
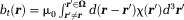

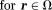
. Background susceptibility sources generate the background field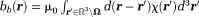 for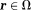. Together, they form a total field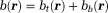 in tissue.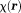 is tissue susceptibility when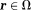.

**Figure 4:**
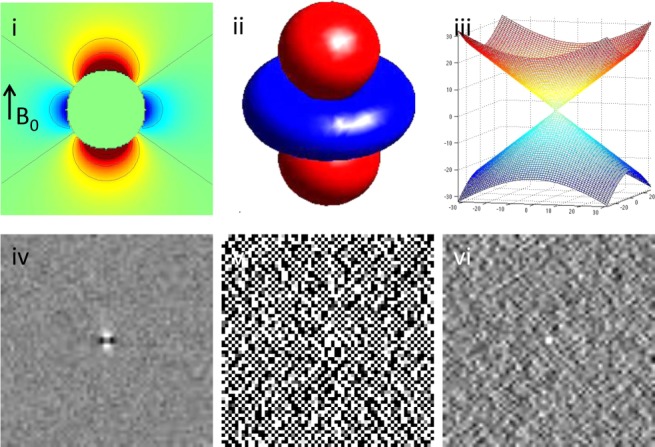
The ill-posedness of the dipole inverse problem. The unit dipole field in sagittal section (i) and its surface rendered contour (ii). (iii) The zero cone surfaces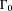 of the dipole kernel in *k*-space. (iv) Field map derived at signal-to-noise ratio (SNR) – 20 induced by a point source. (v, vi) Susceptibility in image space obtained by truncated *k*-space division with the threshold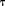 – 0 and 0.1. As a consequence of the dipole kernel zero behavior in the cone surface neighborhood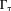, there is substantial noise propagation from the field measurements into the susceptibility estimate (40), as illustrated in an example of reconstruction by direct division (v and vi). A little noise added in the phase map (peak SNR – 20) leads to a totally corrupted susceptibility image that bears no physical resemblance to the true susceptibility source.

The second fundamental challenge is the zero cone surface in the dipole kernel in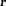-space or in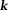-space: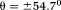 with respect to the main magnetic field (magic angle) (Fig. 4) 14. The dipole kernel in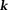-space is nearly flat (with all derivatives vanishing) at the ends of the cone neighborhood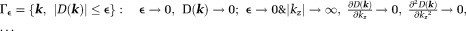, implying that the standard analysis cannot be used to define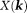 in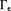 66. Noise in the data would allow many possible susceptibility solutions that differ by an arbitrary amount in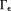 for a given field map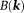, causing a substantial dipole kernel null space. Therefore, the magnetic field-to-susceptibility inverse problem is ill-posed and lacks a unique solution 21.

### QSM Step 1: Background Field Removal

To address the first QSM challenge of the lack of MRI signal in the background regions with susceptibility sources, prior knowledge is needed. The background field may be regarded as slow varying and can be removed by high-pass filtering (HPF). Unfortunately, HPF erroneously removes the low spatial frequency components of the tissue field and fails to remove the high spatial frequency components of the background field near the tissue border 3,67,68, causing substantial errors in QSM. Better priors on the separation between the background and tissue fields are required for accurate determination of the tissue field, such as approximate orthogonality in the projection onto dipole fields (PDF) method 69 or the harmonic property in the sophisticated harmonic artifact reduction on phase data (SHARP) method 70. These priors are systematically organized in the following as solutions to Maxwell’s Equations (Eq. 3).

**Figure 5:**
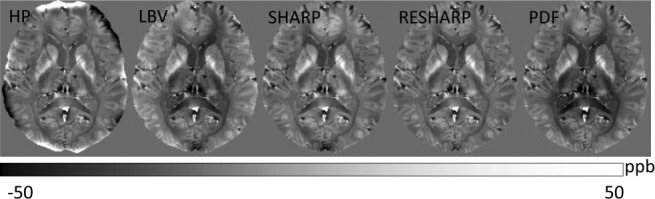
Background field removal using various algorithms. The tissue fields in a healthy volunteer estimated using HPF, LBV, SHARP, RESHARP, and PDF methods, respectively, demonstrate similar tissue patterns but with slight and different accents. HPF, high-pass filtering; LBV, Laplacian boundary value; PDF, projection onto dipole fields; RESHARP, regularization enabled SHARP; SHARP, sophisticated harmonic artifact reduction on phase data.

By definition, the background field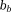 has no source in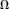, whereas the local tissue field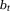 has sources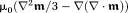 inside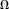 (Eq. 3). From electrodynamics 5 or partial differential equation 71, for a finite domain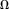, a unique solution to Laplace’s equation Eq. 3 can be obtained according to the values at the boundary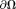 5,72. In MRI, the tissue field is typically weak. At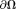, the background field may be approximated as the total field. This is the Laplacian boundary value (LBV) method (Fig. 5) to estimate background field 73.

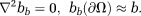
8

Eq. 8 can be solved numerically as a system of linear equations by expressing the Laplacian with a difference operator 74: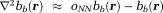. Here,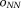 denotes the nearest neighbor average operator. It is advantageous in SNR to express Eq. 8 using the spherical mean value (SMV) operator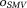 5,70,75: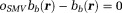. The system matrix is diagonally dominant (with diagonal elements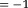), and the Jacobian iterative method can be used 74 to give the (n+1)^th^ iteration solution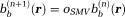. With an initialization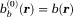 that satisfies the boundary value condition in Eq. 8, the background field can be obtained numerically by repeatedly applying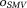, which is the iterative spherical mean value (iSMV) method 76. However, iSMV is slow to converge. Eq. 8 can be efficiently solved using the full multi-grid solver that first finds the solution on a coarse grid and then successively refines the solution on finer grids 74.

When the boundary condition is not known, a partial differential equation in difference form may be regarded as an ill-posed problem, which can be solved by regularization. One regularization is to impose spectral truncation when evaluating the inverse Laplacian (therefore altering the inverse Laplacian),

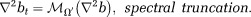
9

Here,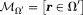 requires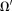 is smaller by a border layer than the available tissue region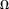 for computing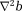. Eq. 9 can be solved as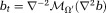: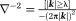, using truncated singular value decomposition (TSVD) (with a carefully chosen truncation value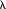) in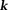-space, and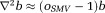, using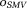 for possible denoising benefit. This is the sophisticated harmonic artifact reduction for phase data (SHARP) 70.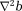 in the border layer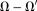 may be recovered using a continuity assumption as in harmonic (background) phase removal using the Laplacian operator [HARPERELLA 62]. Another regularization is the minimal norm of the tissue field (implicitly modifying inverse Laplacian) 62,77:

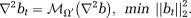
10

Here,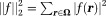 for any field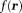 (squared L2 norm). This method is called regularization-enabled SHARP (RESHARP) 77. Eq. 2 can be expressed in a Lagrangian form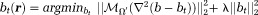.

There is another approach to estimate the background field based on the equivalent source or charge simulation method 78. The background field can be represented by fields of dipoles outside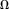 that are approximately orthogonal to the fields of dipoles inside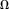 except near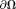. Then the background field can be estimated by all possible PDFs 17,22,69:

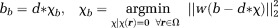
11

Here, noise-weighting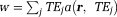 is the phase SNR, which is assumed to be large by linearizing the signal model Eq. 5. Note that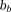 in tissue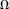 is unique, although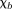 in background
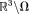
is not. This PDF method may provide a slight advantage in dealing with noise in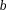 by avoiding the inversion of the Laplacian in Eq. 8 through Eq. 10, and by extending to the nonlinear form, as in Eq. 6 20. However, the orthogonality between fields of dipoles breaks down near the boundary, making PDF prone to over-fitting errors near the border.

Because background and tissue fields are approximately orthogonal, the minimal norm of the tissue field in Eq. 10 is similar to the dominance of the background field at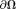 in Eq. 8, making RESHARP similar to LBV. The minimization in Eq. 10 is similar to that in Eq. 11, without noise weighting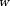, making RESHARP similar to PDF. Therefore, all four methods (LBV, SHARP, RESHARP, and PDF) based on Maxwell’s equations in Figure 5 provide similar performance, whereas there are very strong values near the tissue boundary in the HPF processed tissue field. The assumption or regularization in any method may contain errors, which propagate through into errors in the final reconstructed tissue susceptibility, a challenge for future research.

### QSM Step 2: Field-to-Susceptibility Inversion

To address the second fundamental challenge in QSM, the ill-posedness caused by the dipole kernel zeroes, prior knowledge is again needed to uniquely identify the susceptibility component in the dipole kernel null space. For simplicity, we consider the high SNR case for which the phase noise is approximately Gaussian with variance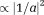 [and we can extend to the general SNR case using the complex signal as in Eq. 6 20]. Then Eq. 5, after background field removal, is reduced to a linear field-to-susceptibility inverse problem (Eq. 2 with noise),

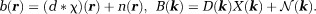
12

Arbitrary susceptibility values in the dipole zero cone neighborhood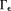 are allowed in Eq. 12. A regularization can be used to specify susceptibility values in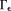. Alternatively, “missing data” about the susceptibility in one orientation can be recovered by reorienting the subject in a fixed magnet and resampling the MRI signal 14,79. This method is known as the calculation of susceptibility using multiple orientation sampling (COSMOS), which delivers an exact reconstruction by fully sampling the susceptibility 14,22. Unfortunately, acquiring multiple scans of patients in different orientations is not clinically acceptable. We should focus on the regularization approach to QSM using single orientation data.

Regularizations can be expressed in various mathematical forms including TSVD and assumptions of a smooth, sparse, or piece-wise smooth solution. Consequently, there are too many QSM methods to be characterized by a unifying framework. For the purpose of illustrating concepts, we outline two important classes of QSM methods: 1) the closed-form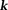-space approach, exemplified by TSVD based on matrix computation 80, and 2) the Bayesian approach based on optimization 52, also known as the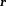-space approach. Specific algorithm formulas, codes, and results are summarized in the next section on experimental validation. All of these solutions may suffer from model errors when the mathematical properties are not consistent with the physical reality 7,10,11,17,21,81. Additionally, noise will always propagate into the final solution.

Closed-form solutions form a class of noniterative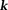-space methods. One example is TSVD 82: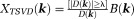 with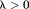, which is similar to a Tikhonov-regularized minimal norm (MN) solution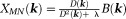 21,22. More commonly used in QSM is a TSVD variant called truncated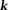 -space division (TKD) 10:

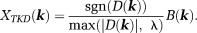
13

Truncation obviously leads to an underestimation of the susceptibility values in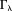, and consequently produces streaking artifacts along the magic angle in the susceptibility map. This error in the TKD method is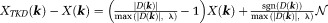: the first part is the regularization error from points in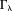 where the kernel has been modified by truncation; the second part is the noise error from all data points in k-space, but interestingly is also dominated by points in and near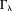. The underestimation in Eq. 13 may be compensated by a scale factor 26. The streaking in Eq. 13 may be reduced using a high threshold [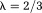, susceptibility – field convolving with a kernel 26] or using iterative filtering (defined by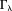 in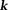-space) of signals outside high-susceptibility structures in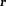-space defined by a mask [iterative Susceptibility Weigted Imaging and susceptibility Mapping (iSWIM), 30]. A variant of MN is a Tikhonov-regularized minimal gradient norm [29: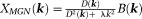].

Bayesian regularizations form a class of iterative optimization methods. To allow optimal treatment of regularization error 83, prior information is regarded as a probability distribution function (pdf) typically expressed as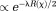: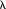 is a tunable regularization parameter and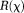 is a functional of the susceptibility map. Noise is also characterized by its pdf. Then, the optimal solution with minimal total error from regularization and noise is the maximum a posteriori (MAP) estimate 84. For Gaussian noise with pdf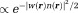 (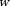 as in Eq. 11) in the estimated field, the posterior probability is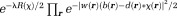, the maximization of which is the MAP solution:

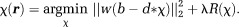
14

The first term in Eq. 14 is the weighted data fidelity term, which contains noise; the second term is the regularization term, which contains the regularization error. The regularization parameter,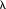, may be chosen to provide a minimal total error in a given imaging situation, and is typically chosen such that the regularization error is approximately equal to the expected noise level, a criterion known as the discrepancy principle 85–87.

**Figure 6:**
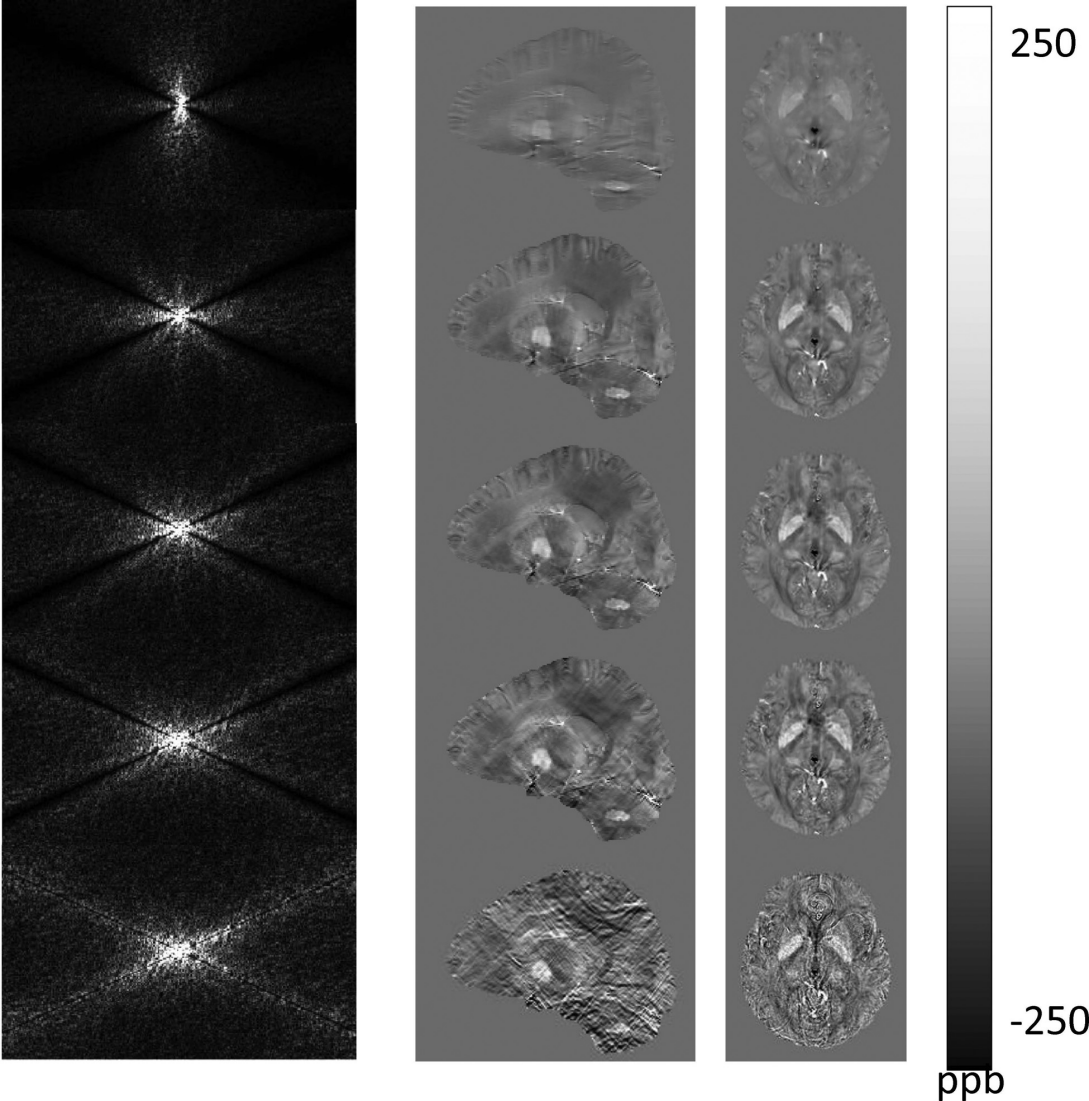
Evolution of susceptibility solutions in conjugate gradient. Susceptibility images in *k*-space (left column) and *r*-space after the first, third, fifth, 10th, and 100th iterations using the conjugate gradient solver demonstrate that the none-cone region in *k*-space converges quickly in the first few iterations; and the later iterations mainly contribute to signal in the cone region that manifests as streaking artifacts in the sagittal view and noise in the axial view in *r*-space.

There are connections between Bayesian optimization and noniterative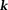-space methods. If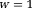 in Eq. 14, then L2 norm-based Tikhonov regularization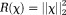 leads to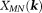 and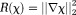 leads to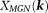; both are noniterative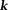-space methods. Because MRI phase noise requires spatially varying weighting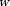, noniterative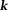-space methods may suffer from noise errors 88. A wide range of forms for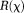 in Eq. 14 have been reported, including piece-wise constant susceptibility 11,89, smooth susceptibility or susceptibility gradient 21, sparse susceptibility gradient or wavelet 21,27,90, and morphological consistency of the susceptibility map 17,19,21,25, some of which are detailed in the next section. We should note the property of the conjugate gradient method that typically is used to minimize the data fidelity term in Eq. 14 as in LSQR: The solution is initialized as zero by default, points outside the zero cone neighborhood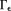 are calculated firstly (and properly) according to Krylov sequence, and later iterations fill structured noise in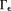 that cause streaking artifacts 16,52,91 (Fig. 6). Use of a small iteration number (n – 5) may be regarded as an implicit regularization for a solution with moderate streaking and zero value at the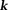-space center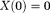 16,88. For a solver of Eq. 14 that includes use of LSQR, such as in the Gauss-Newton method, its final solution may have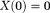. Eq. 14 may only determine the susceptibility up to a constant [similar to the Neumann boundary value problem for Laplace’s equation 5]. Specific solvers may introduce implicit regularization in their output. A reference to water, such as the cerebrospinal fluid in the ventricles, may be used for consistent display of susceptibility values in QSM.

Available anatomic information in a specific imaging situation defines a physical prior for morphology-enabled dipole inversion (MEDI) 19,83,92. The edges in the desired susceptibility map are likely to be colocated with edges in known anatomical images, because they reflect the same anatomy. The colocalization of edges may be expressed as the sparsity of susceptibility edges outside the known edge locations using the L0-norm or the more manageable L1-norm 93. This minimizes streaking artifacts common to the dipole kernel nullspace. From Eq. 14, one MEDI implementation to reconstruct QSM can be formulated as 17,19:

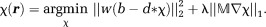
15Here,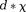 is evaluated in Fourier space by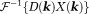,
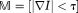
for anatomic image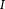 and threshold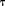 (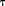 is chosen such that approximately 30% of voxels are labeled as edges), and
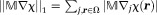
(L1 norm). Because the dipole kernel (Fig. 4), similar to a HPF, preserves susceptibility edges in the tissue field image 5 and accordingly in the T2*-weighted (T2*w) magnitude image, the GRE magnitude 17 and phase images 25 can be used as the anatomic images. The nonlinear Eq. 15 can be solved using the Gauss-Newton method 52 without explicit formation of the memory costly inverse Hessian matrix 19. The data fidelity term in Eq. 15 can be generalized to Gaussian noise in complex data (e.g., Eq. 6) and can be solved using a procedure identical to Eq. 15 20.

New priors continue to be proposed; the search for the best prior is ongoing. An optimal prior may be specific to the imaging application. There has been a preliminary attempt to compare several priors 88. Multicenter trials are needed to establish a consensus on application-specific priors, which leads to the topic of the next section.

### QSM Source Codes and Experimental Validations

To enable readers to try QSM algorithms on their own, here we organize the available MATLAB (MathWorks) codes for certain QSM algorithms, along with testing data (http://weill.cornell.edu/mri/pages/qsm.html). We tried to consider all the QSM methods that were published before December 1, 2013, classifying similar methods into the same category. We asked the first or corresponding authors to share MATLAB (MathWorks) codes of their methods, proofread our implementations, and comment on results of testing data, and we thank them for their valuable feedback. The page limitation forced us to select one (perhaps the most widely used) of multiple algorithms published by each group, leading to the following seven methods in Table 1: TSVD 22; TKD 10; iSWIM 30; MEDI 19; compressed sensing compensated (CSC) inversion 27; homogeneity-enabled incremental dipole inversion (HEIDI) 25; and total variation using split Bregman (TVSB) 28. The first two methods do not require anatomic prior information. The third method, iSWIM, incorporates an anatomic prior iteratively into the ***k***-space approach. The last four methods are based on the Bayesian approach and are listed in chronologic order. The Bayesian methods all aim to minimize a function consisting of a data fidelity term in the L2 norm (measuring noise power) and a regularization term in the L1 norm or total variation (promoting sparsity). Whereas MEDI and HEIDI attempt to sparsify the edge difference with known anatomical priors, CSC promotes image sparsity in the wavelet domain, and TVSB hugely accelerates reconstruction speed by dropping noise whitening.

**Table 1:** Accuracy Assessments via Linear Regression Based on Voxel Values Between Methods and Reference Standards (Truth for Simulated Brain, Prepared Concentration for Gadolinium Phantom and COSMOS for In Vivo Brain) as well as Recon Time for In Vivo Brain Imaging

	Simulated Brain		Gadolinium Phantom		In Vivo Brain		
	Slope	R^2^	Slope	R^2^	Slope	R^2^	Time (sec)
TSVD	0.83	0.83	0.76	0.99	0.80	0.45	1.7
TKD	0.91	0.89	0.82	0.99	0.88	0.34	1.8
iSWIM	0.81	0.82	0.84	0.99	0.81	0.48	14
MEDI	0.99	0.99	0.92	1.00	0.93	0.59	1008
CSC	0.63	0.65	0.87	0.99	0.79	0.60	3463^(1)^
HEIDI	0.82	0.90	0.82	0.99	0.80	0.55	715^(2)^
TVSB	0.83	0.94	0.87	1.00	0.84	0.42	40

All the calculations were performed on a PC equipped with Intel® Core i7–3770k CPU @ 3.5 GHz and 32 GB of memory, except 1 was performed on a personal laptop with Intel Core i5-M2450 CPU @ 2.5 GHz and 8 GB of memory, and 2 was performed on a PC with Intel Core i5–2320 CPU @ 3.00 GHz and 16 GB of memory.

CPU, central processing unit; COSMOS, calculation of susceptibility using multiple orientation sampling; CSC, compressed sensing compensated; GB, gigabytes; GHz, gigahertz; HEIDI, homogeneity-enabled incremental dipole inversion; iSWIM, iterative susceptibility weighted imaging and susceptibility mapping; MEDI, morphology-enabled dipole inversion; PC, personal computer; TKD, truncated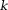 -space division; TSVD, truncated singular value decomposition; TVSB, total variation using split Bregman.

Rigorous experimental validation with a reference standard is required to assess the accuracy of any quantitative technique. This is particularly necessary for a technique involving regularization such as the QSM algorithms in Table 1. We performed three validation experiments: 1) numerical simulation with known truth, 2) MRI data of gadolinium phantom with known susceptibilities, and 3) in vivo brain MRI with susceptibilities assessed by COSMOS 92,94. These validations were imperfect, particularly because COSMOS contains errors from noise, orientation registration, and WM susceptibility anisotropy. However, they can serve as a starting point for readers to experience various QSM methods. Details of the experimental setup are described in Validation data in the supporting information. Although evaluations here are not intended to be conclusive, they allow readers to assess various QSM methods by their qualities (by examining the streaking artifacts), accuracies (by voxel-based linear regression with ground truth or reference), and computing costs (by taking the median running time of 5 consecutive runs).

**Figure 7:**
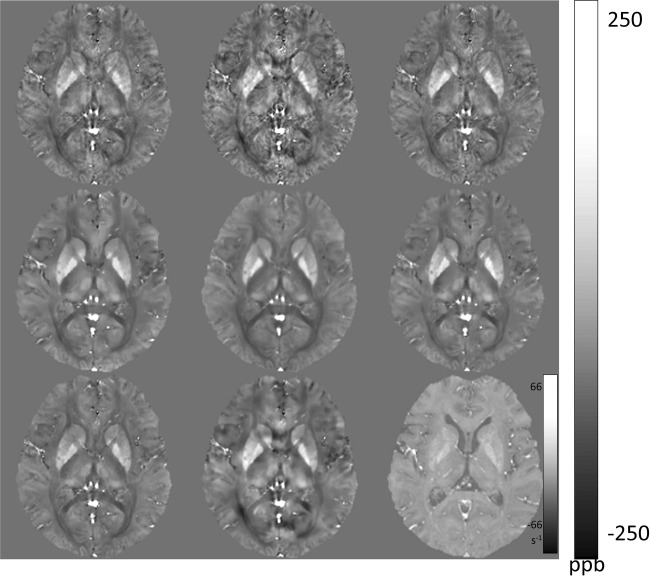
Comparison of various quantitative susceptibility mapping reconstruction methods. QSM images are reconstructed using various methods from left to right and then top to bottom: TSVD, TKD, iSWIM, CSC, COSMOS, MEDI, HEIDI, TVSB, and R2* map. Most similar to COSMOS are MEDI, CSC, and HEIDI, with only very subtle differences among them: CSC has less black dots; MEDI has better defined dorsomedial nuclei of thalamus. CSC, compressed sensing compensated; COSMOS, calculation of susceptibility using multiple orientation sampling; HEIDI, homogeneity-enabled incremental dipole inversion; MEDI, morphology-enabled dipole inversion; QSM, quantitative susceptibility mapping; TKD, truncated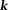-space division; TSVD, truncated singular value decomposition; TVSB, total variation using split Bregman.

The numerical simulation (Fig. S1a,b in the supporting information) demonstrated that all methods yielded satisfactory image quality, with minimal streaking in the sagittal view. The phantom experiment (Fig. S2a–c in the supporting information) demonstrated that constraining solutions’ energies at the cone region alone was not sufficient to suppress streaking (TSVD, TKD). The iSWIM method reduced streaking by iterative filtering, and the most significant improvement was observed when spatial constraints were incorporated during dipole inversion (MEDI, CSC, HEIDI, TVSB). The in vivo brain MRI (Fig. 7) demonstrated that all methods successfully generated QSMs. The major structures containing high levels of ferritin (basal ganglia and nuclei in deep gray matter) or deoxyhemoglobin (veins) are shown with high paramagnetic values on QSM, and WM tracts are shown with diamagnetic values on QSM. Rapid streaking signal variations not on COSMOS were observed in TSVD and TKD, likely artifacts originating from veins. The iSWIM method reduced but did not eliminate these artifacts. TVSB overblurred compared to COSMOS; one of the causes may be its lack of noise whitening. MEDI, CSC, and HEIDI yielded QSM images similar to COSMOS.

In the accuracy assessment (Table 1), MEDI and TVSB achieved the best slope and coefficient of determination (R^2^) in both numerical simulation and phantom experiments, presumably because the piece-wise constant nature of the susceptibility distribution matched the assumptions in MEDI and TVSB. In the human brain, although MEDI provided the highest slope, none of the methods provided adequate R^2^ values. A possible cause may be voxels of WM with susceptibility anisotropy. The reconstruction speed of the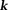-space methods were much faster than that of the iterative Bayesian methods (Table 1). However, the split Bregman implementation in TVSB showed promise of fast online reconstruction for the Bayesian methods.

### Clinical Applications Under Development

QSM has become sufficiently accurate for measuring the strong susceptibilities of biomaterials, including iron distribution (ferritin), in the deep brain nuclei and basal ganglia; deoxyhemoglobin in the veins; blood degradation products (hemosiderin in late stage); calcification (hydroxylapatite crystals); and exogenous species such as gadolinium. Clinical applications of QSM are being developed to probe neurodegenerative and inflammatory diseases, to assess hemorrhage, to measure metabolic consumption of oxygen, and to guide and monitor therapy. QSM can also remove blooming artifacts in traditional T2*w imaging 95, providing an accurate definition of the distribution of magnetic biomaterials in MRI. In this brief survey, we focus on neurological applications, although applications outside the brain are also promising 96.

### Diamagnetic Biomaterial-Based Applications

**Figure 8:**
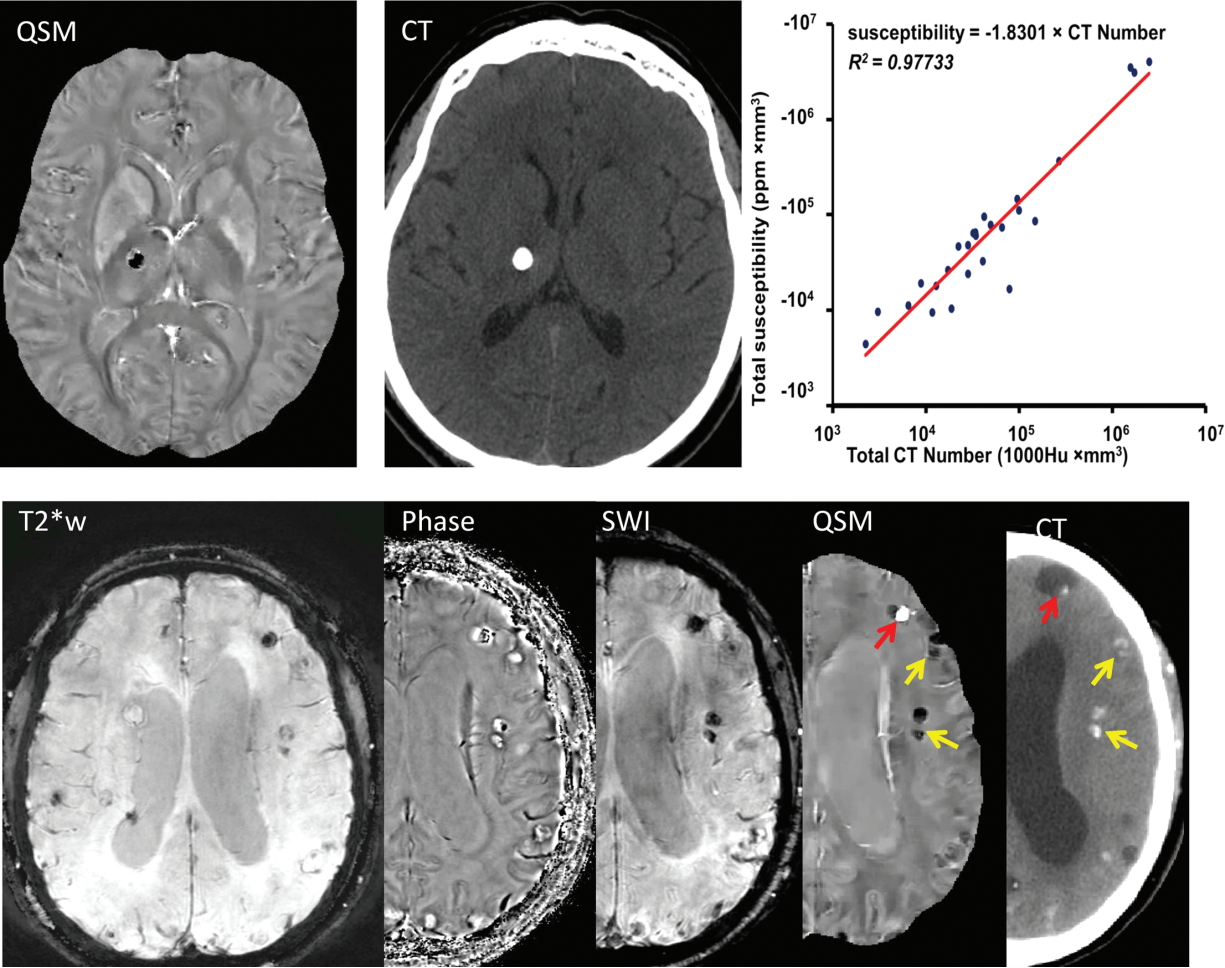
Quantitative susceptibility mapping for measuring diamagnetic biomaterials such as calcifications. Top row: susceptibility of calcification measured on QSM (dark oval in right thalamus) demonstrates very good linear correlation with Hounsfield units measured on CT. Twenty six patients (64 calcifications) were included in the linear regression. Bottom row: Neurocysticercosis in T2* weighted, magnitude, phase, SWI, QSM, and CT images has both calcified and active lesions. Among all MRI images, only QSM shows the active lesion with positive susceptibility (red arrow) and clearly show the calcified lesions with negative susceptibilities (yellow arrows). The CT section is slightly tilted from the orientation of the MRI section, causing a discrepancy in the lesion’s appearances. QSM, quantitative susceptibility mapping; SWI, susceptibility weighted imaging. Source: Chen et al, Radiology 2014;270:496–505.

QSM can easily differentiate diamagnetic calcification from paramagnetic materials such as hemosiderin 89,97. Both calcification and chronic hemorrhage appear hypointense on GRE magnitude images and may be undetectable on conventional T1- and T2-weighted SE imaging 98,99. GRE phase imaging has long been recognized for its ability to identify diamagnetic calcifications 100,101, but there is no study demonstrating its diagnostic accuracy. As such, CT is widely used for detecting calcifications despite its use of ionizing radiation 102. QSM may replace CT for detecting calcification in neurocysticercosis (Fig. 8) and tumors 103–106. A clinical study demonstrated that QSM is superior to phase imaging and has a very high sensitivity (90%) and specificity (95%) for the detection of intracranial hemorrhage and calcification 97. QSM can also be used to measure the loss of myelin 107, another important diamagnetic biomaterial.

### Paramagnetic Heme Iron (Deoxyhemoglobin, Metahemoglobin, Hemosiderin)-Based Applications

**Figure 9:**
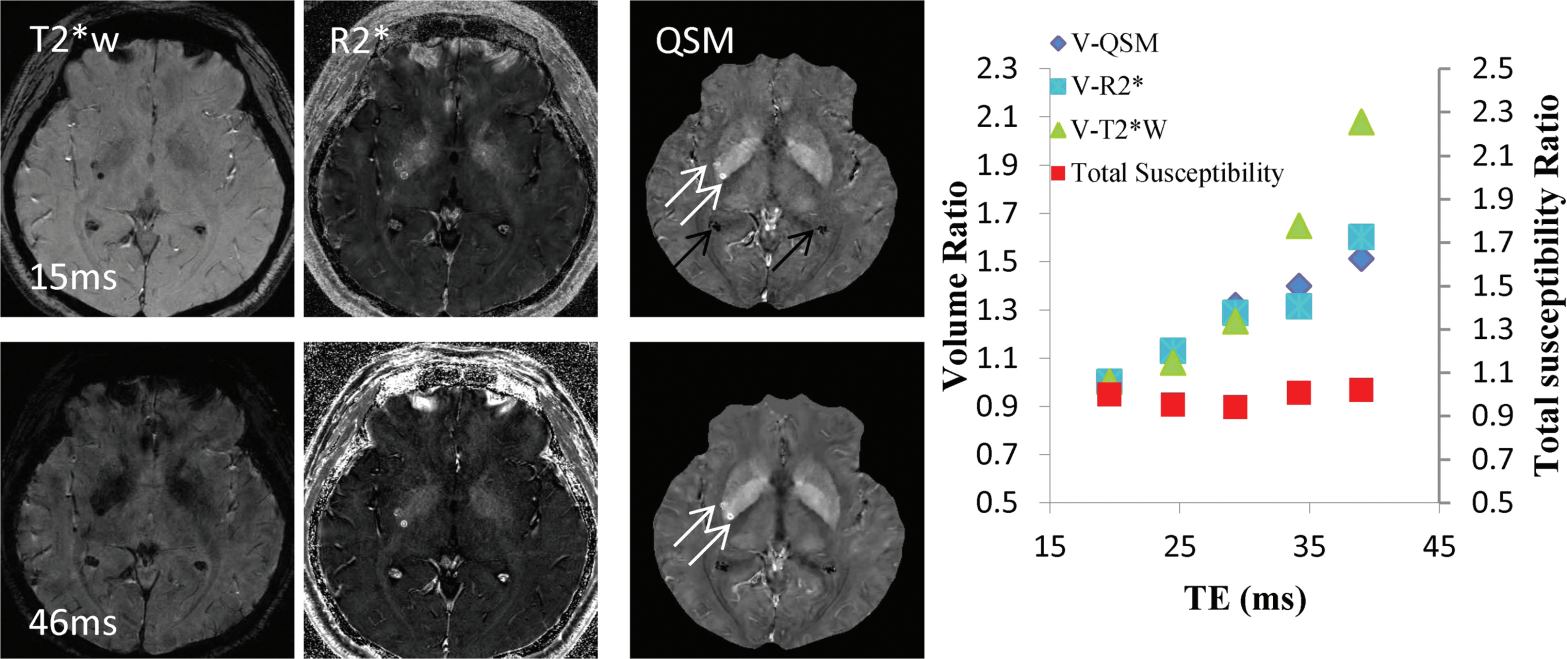
Quantitative susceptibility mapping for measuring paramagnetic heme iron. The total susceptibility of a cerebral microbleed measured on QSM is a physical property that is independent of TE, providing a universal measure for cerebral microbleeds burden (10 patients with 40 microbleeds). Left image panel: Microbleed appearance changes with TE (15 msec top row; 46 msec bottom row) drastically in magnitude and moderately in the R2* map but little in QSM (white arrows). Ventricle calcifications have the same sign on T2*w and R2* but opposite sign on QSM (black arrows) as microbleeds. Right graph: When TE was increased from approximately 20 to 40 msec, the measured cerebral microbleed volume increased by mean factors of 1.49 ± 0.86 (standard deviation), 1.64 ± 0.84, and 2.30 ± 1.20, respectively, for QSM, R2*, and T2*w, respectively (P < .01). However, the measured total susceptibility with QSM did not show significant change over TE (P – .31), and the variation was significantly smaller than any of the volume increases (P < .01 for each). QSM, quantitative susceptibility mapping; TE, echo time. Source: Liu et al, Radiology 2012;262:269–278.

During blood degradation in hemorrhage (Fig. 1b), susceptibility progressively increases from oxyhemoglobin (diamagnetic) to deoxyhemoglobin (paramagnetic with 4 unpaired electrons in Fe^2+^), methemoglobin (strongly paramagnetic with 5 unpaired electrons in Fe^3+^), and hemosiderin (super paramagnetic with possible magnetic domain formation or ferromagnetic) 108–111. GRE is more sensitive than CT at detecting intracerebral hemorrhage 112,113. However, the T2* hypointensity in GRE suffers from blooming artifacts that are highly dependent on imaging parameters. Reliably estimating the hematoma volume is critical for managing hemorrhagic stroke patients 114,115, but it is difficult to do on GRE 95. QSM removes these blooming artifacts 17,19,20 and can be used as a universal measurement of microbleeds (Fig. 9) and hematoma volume in GRE MRI 111,116,117.

Susceptibility values in QSM can be converted to the venous deoxyhemoglobin concentration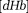 according to deoxyhemoglobin’s molar susceptibility
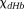
–10765 ppb 48,118–120, allowing quantitative fMRI 117. The tissue metabolic rate of oxygen consumption (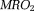) is regarded as a fundamental biomarker for assessing viability of aerobic tissue such as those in the brain, heart, and kidney 121. Measurements of regional blood flow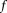 by quantitative perfusion, such as the arterial spin labeling 122 and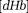 by QSM, can be used to map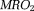 according to oxygen mass conservation 123–126: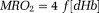. For tissues with nonheme iron such as ferritin, susceptibility contributions can be corrected using iso-metabolism manipulation 127.

### Paramagnetic Nonheme Iron (Ferritin)-Based Applications

Iron overload can generate biologically toxic reactive oxygen species, causing oxidative stress and damaging macromolecules including proteins, lipids, and DNA 128,129. Excessive iron deposition in specific regions of the brain has been observed in many neurodegenerative diseases 130–133, including Parkinson’s disease, Alzheimer’s disease, amyotrophic lateral sclerosis, Huntington’s disease, Friedreich’s ataxia, and multiple sclerosis 134–137. Consider the example of Parkinson’s disease (PD). Several studies demonstrate an increase of iron deposits in the substantia nigra in PD 138–142, perhaps increasing with disease progression 143. The abnormal increase in nigral iron generates reactive oxygen species 131, possibly causing nigrostriatal dopamine neuron degeneration 144,145 and alpha-synuclein aggregation 146,147. MRI R2* (1/T2*) has been used to measure tissue iron content 9,148–153, but R2* in a voxel reflects the field variance within that voxel (see definition after Eq. 5). The latter depends on the background field, surrounding tissue susceptibilities, and imaging parameters including field strength, voxel size, and TE. The MRI phase has also been used to measure brain iron in PD 9,154–159, but phase is a convolution of tissue susceptibility in space (Eq. 2). QSM overcomes the problems of R2* and phase images, enabling reliable iron quantification when there is no other substantial susceptibility contributor 90,132,133,142,160–162. Perhaps for this reason, QSM has been widely used to measure brain susceptibility 94,163.

QSM can improve visualization of the target in deep brain stimulation (DBS), the surgical implementation of stimulating electrodes in the subthalamic nuclei (STN), or the globus pallidus pars interna (GPi) for treating neurological disorders including PD 164–167. The anatomical accuracy of electrode lead placment is critical for a successful surgical outcome 168–176. Intraoperative CT, typically used to guide DBS, has poor contrast for the basal ganglia structures 170,172,177–181. MRI provides better tissue contrast than CT, but the visualization contrast for STN and GPi is still poor in standard T2-weighted imaging, and high field MRI using T2*w imaging has been sought for DBS 182–186. QSM can be used to remove the blurring present in T2*w and improve contrast-to-noise-ratio by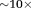 for the visualization of the STN 160,161,187 (Fig. 0).

**Figure 10:**
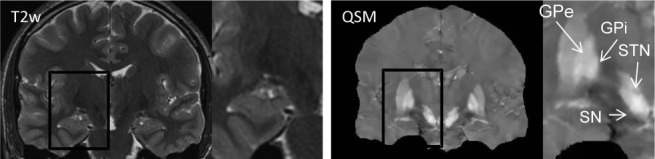
Quantitative susceptibility mapping for visualizing deep brain stimulation targets. Globus pallidus interna (GPi) and subthalamic nucleus (STN), surgical targets for deep brain stimulation, are either invisible or inseparable from surrounding tissues on T2W image (with zoom), but are clearly visualized on deep brain stimulation (QSM) (with zoom). Other basal ganglia structures well-defined on QSM include globus pallidus pars externa (GPe) and substantia nigra (SN). Source: Liu et al, Radiology 2013;269:216–23.

### Paramagnetic Contrast Agent Biodistribution Quantification-Based Application

**Figure 11:**
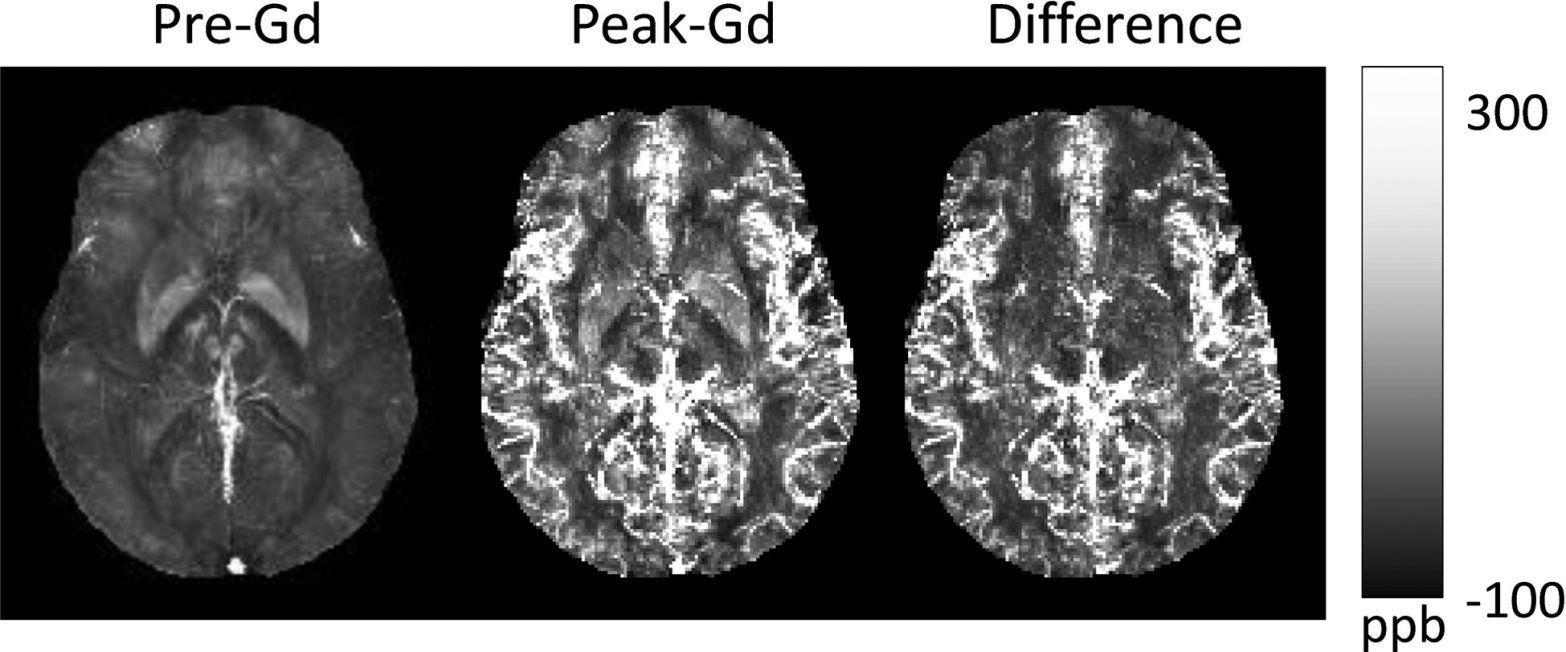
Quantitative susceptibility mapping for quantifying paramagnetic contrast agents. In an in vivo dynamic gadolinium (Gd) enhancement study of the brain, time-resolved Quantitative susceptibility mapping (QSM) was developed using spiral readout and temporal resolution acceleration with constrained evolution reconstruction (TRACER) complex image reconstruction. The difference image divided Gd molar susceptibility generates time-resolved Gd concentration map. Source: Xu et al, MRM 2014, epub.

QSM can be applied to measure the biodistribution of highly paramagnetic contrast agents (CA), providing an effective tool for quantifying CA concentration [CA] in MRI 188,189. The quantitative study of the phase change observed in contrast-enhanced MRA 190 was an initial motivation to formulate the field-to-susceptibility inverse problem 31. Absolute determination of [CA] according to T1/T2 enhancement effects requires calibration and is highly susceptible to flip angle errors 191–193. CAs with limited access to water demonstrate the well-known T1/T2 relaxation quench 191,192,194–197; relaxation enhancement requires CA binding with bound water, which, in turn, exchanges chemically with surrounding bulk water (CA↔bound H_2_O↔bulk H_2_O) 36,198–200 (see Supporting Figure S4). [CA] has no well-defined relationship with R2*. QSM overcomes these problems of mapping [CA] in T1/T2/T2* imaging and may be useful for determining the biodistribution of targeted CAs in molecular MRI. A high temporal-spatial gadolinium concentration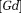 map can be obtained using QSM and fast imaging 201, from which quantitative perfusion map may be generated 50,202 (Fig. 11).

### Mixed Diamagnetic and Paramagnetic Applications

**Figure 12:**
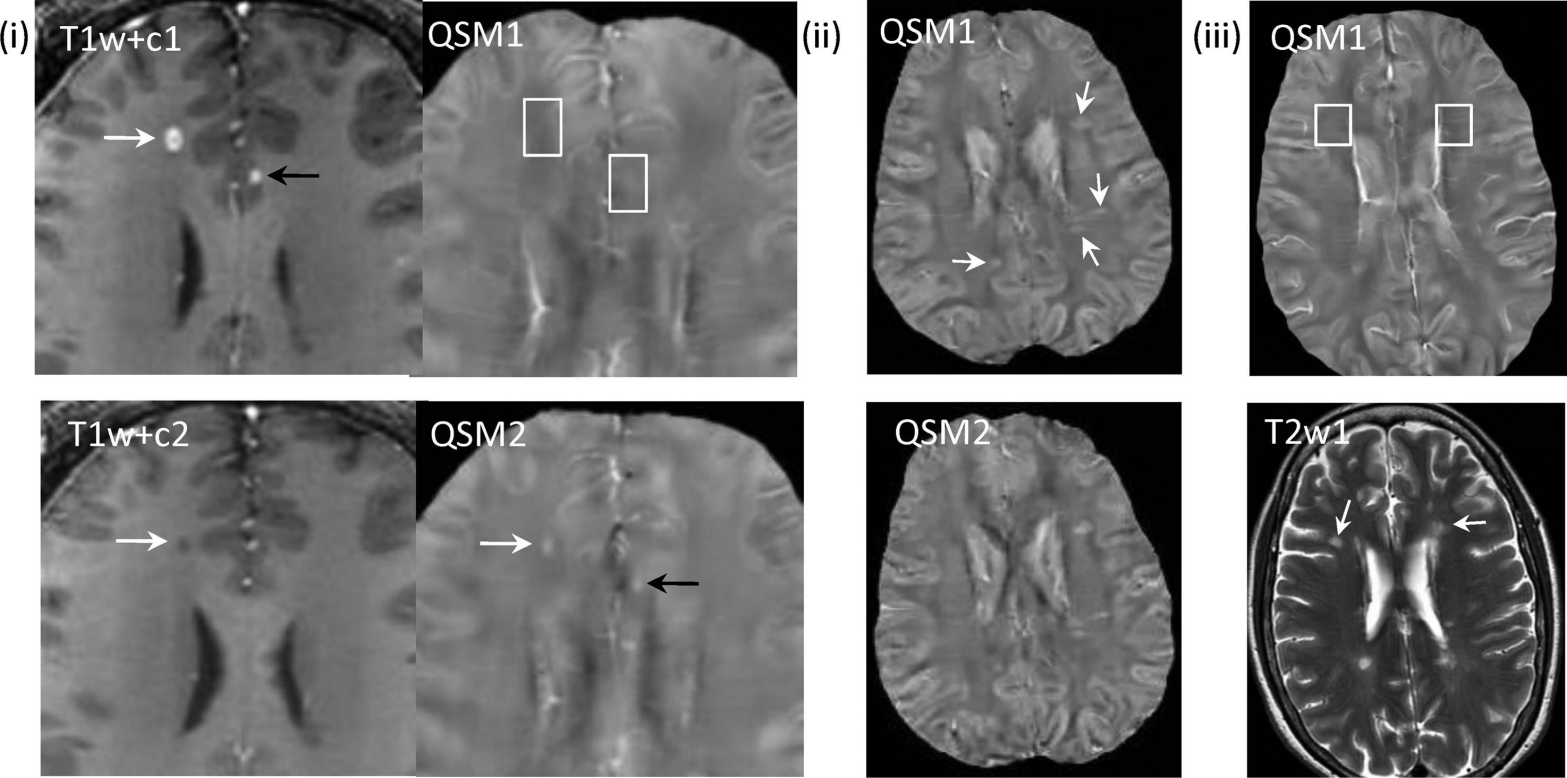
Quantitative susceptibility mapping for quantifying a mixture of paramagnetic and diamagnetic biomaterials. (i) Acute enhancing lesions in a 32-year-old male with RRMS at two time points: initial study (T1w + c1 and QSM1, 1st row) and 3-month follow-up (T1w + c2 and QSM2, 2nd row) (T1w + c – T1-weighted imaging with Gd). Lesions appear in the right frontal WM (white arrows) and in the lcc (black arrows). Both lesions are enhancing (arrows) on T1w + c1 and isointense (white boxes) on QSM1. The lesions changed on follow up to nonenhancing on T1w + c2 and hyperintense on QSM2 (arrows). The right frontal WM matter lesion shrunk between QSM1 and QSM2. The lcc lesion (black arrow) recovered to normal appearing on T2w and T1w (not shown), T1w + c. (ii) Nonenhancing lesions (33y, f, RRMS) on QSM at two time points (2nd row was 6 months later). All QSM lesions at time point 1 were estimated to be 1.2y using prior MRIs. All lesions (arrows) are QSM hyperintense on both time points with similar values. (iii) Chronic nonenhancing lesions (50y f RRMS) on QSM and T2W. Two lesions over 10 years old were detected (white arrows). They appear isointense on both QSM (white boxes, only initial study shown). lcc, left cingulate cortex; QSM, quantitative susceptibility mapping; RRMS, relapsing-remitting multiple sclerosis; WM, white matter. Source: Chen et al, Radiology 2014;271:183–192.

**Figure 13:**
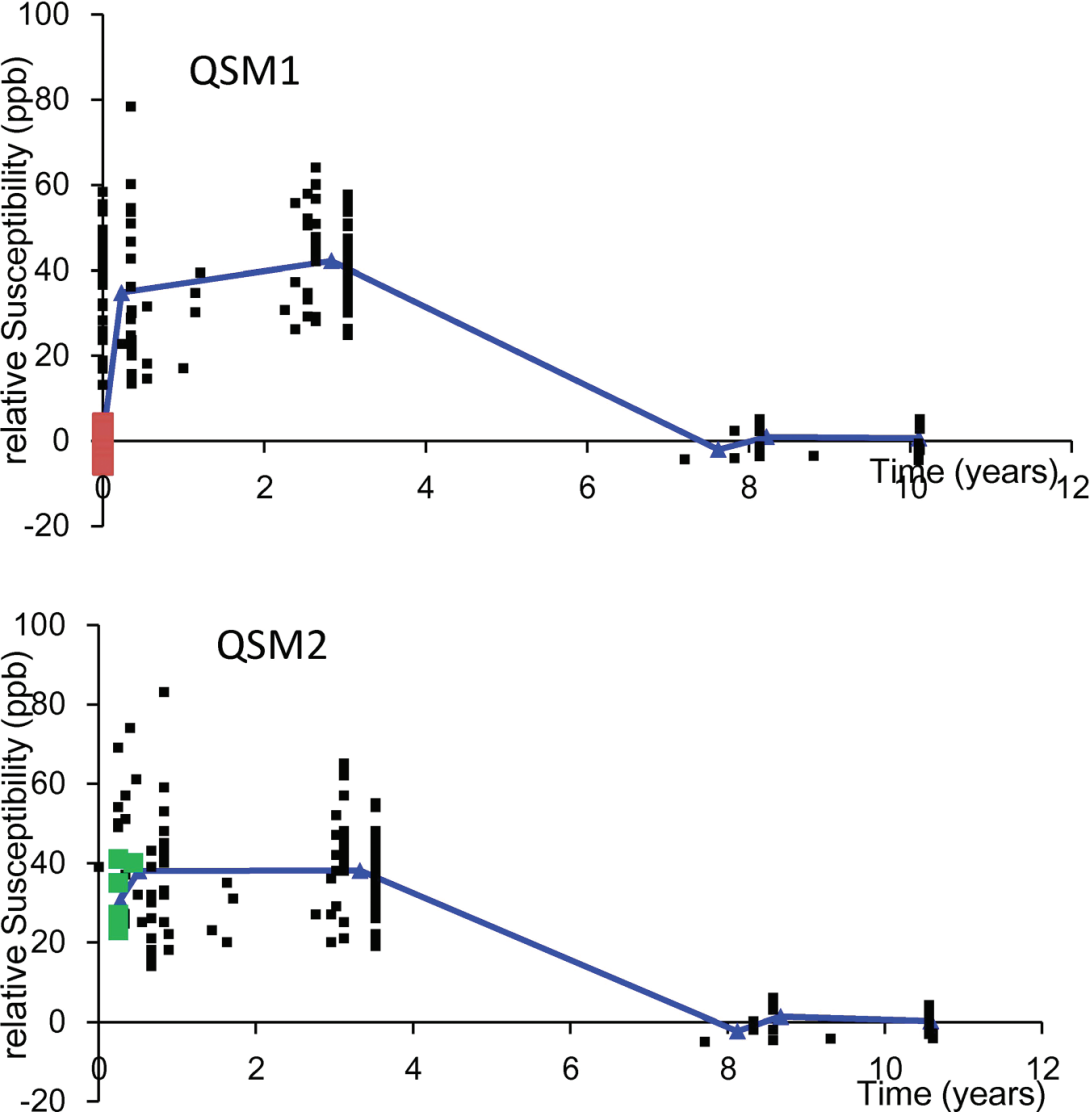
Time course of susceptibilities of multiple sclerosis lesions. The susceptibility time course may provide new insight into pathophysiologic features of MS lesions (23 patients with 162 lesions): Magnetic susceptibility of MS lesion increases rapidly as it changes from enhanced to nonenhanced, attains a high-susceptibility value relative to NAWM during its initial few years (approximately 4 years), and gradually dissipates back to susceptibility similar to that of NAWM as it ages. The graphs depict lesion susceptibility values (relative to NAWM) at various ages in QSM1 performed at a first time point (top) and in QSM2 at a second time point (bottom). Red points in QSM1 denote acute enhancing lesions at lesion age – 0 year; follow-up presented as green points in QSM2 demonstrated a substantial increase in susceptibility. Blue lines represent average susceptibilities of nonenhanced lesions in the age groups of 0 to 2, 2 to 4, 6 to 8, and 8 to 10 years and enhancing lesions. QSM, quantitative susceptibility mapping; MS, relapsing-remitting multiple sclerosis; NAWM, normal appearing white matter. Source: Chen et al, Radiology 2014;271:183–192.

GRE phase images have been used to study iron distribution and demyelination in multiple sclerosis (MS) lesions 203–207. Iron distribution has been reported to be abnormally high in both the basal ganglia and lesions in MS patients 137,203,204,208,209 and may vary with lesion age and inflammatory status 204,210,211. QSM can be used to measure susceptibility changes in both lesions and nonlesion tissues in MS brains 212,213. A recent QSM study of magnetic susceptibilities of MS lesions 214 demonstrates that MS lesion susceptibilities start at the level of normal appearing WM (NAWM) (age – 0y, acute enhancing), quickly increase (within 0.5y) above that of NAWM, remain almost constant for a period (0–4y, intermediately aged and nonenhancing), and then decay gradually back to that of NAWM (> 7y, chronic nonenhancing) (Figs.12 and 13). This MS lesion susceptibility time course is consistent with the no-phase-variation on the 2.5y follow-up of nonenhancing MS lesions seen in another study 215, and with the rapid increase of off-resonance frequency observed in acute enhancing lesions in a third study 216. Investigations are ongoing to connect susceptibility time course and MS cellular activities. QSM may constitute an important new biomarker for the inflammatory and neurodegenerative activities in MS.

### Initial Results in Aorta, Breast, Extremity, and Kidney

**Figure 14:**
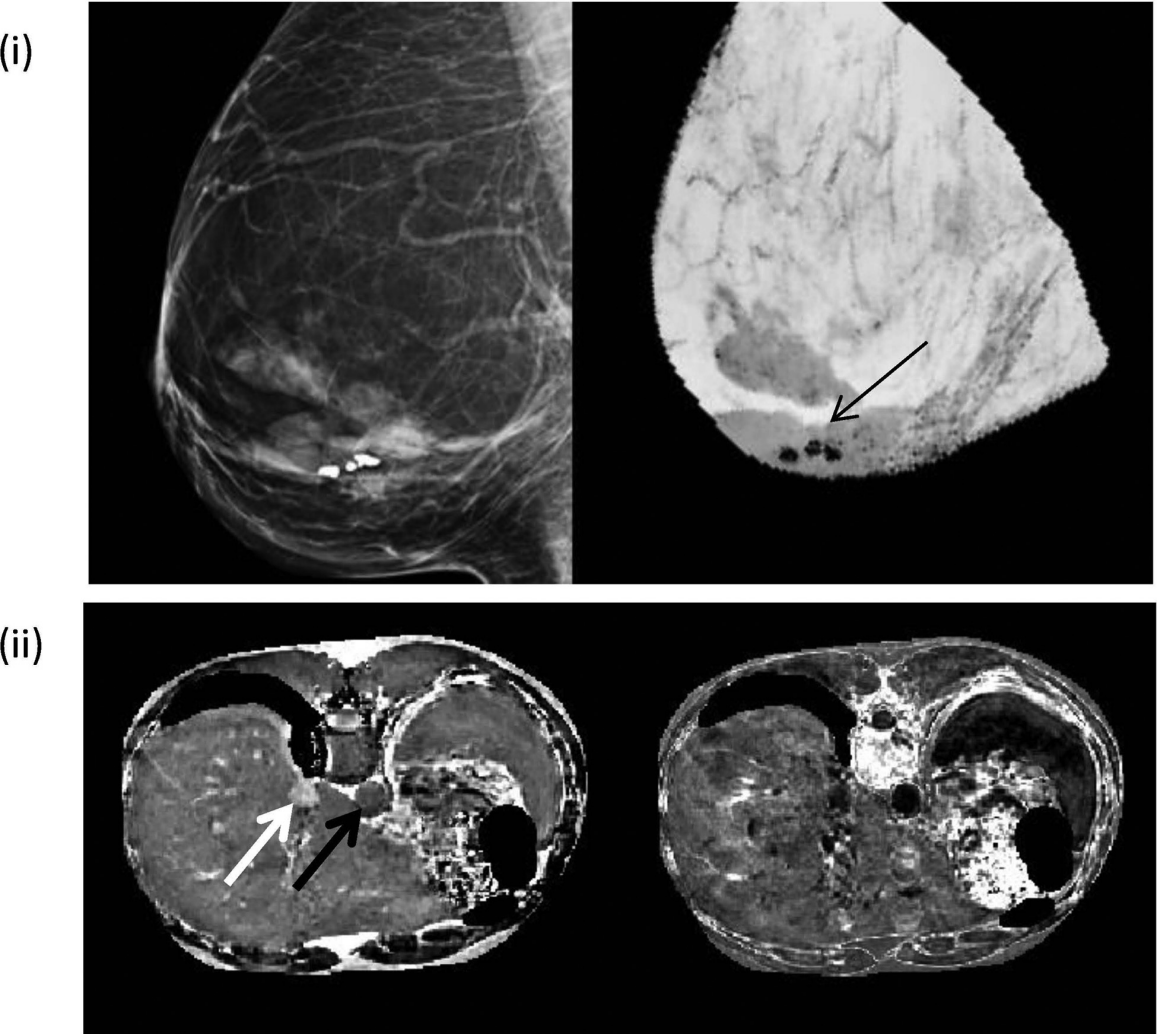
Quantitative susceptibility mapping applications in the breast and liver. (i) Left image is a mammogram and right image is the corresponding QSM (minimal intensity project through the 3D volume) of a breast in a female patient with three calcified nodules (arrow on QSM). Fatty tissues in the breast appear less diamagnetic compared to the gland. (ii) QSM and R2* images of a liver are shown in the left and right, respectively. Hepatic vein and subcutaneous fat (white arrows in left) appear paramagnetic. The susceptibility difference between the hepatic vein (white arrow) and the aortic artery (black arrow) are 0.53 ppm. 3D, three dimensional; QSM, quantitative susceptibility mapping.

QSM applications beyond the brain are also under active development (Fig. 14). The susceptibility values from phase data of the aortic arch during a Gd bolus passage may provide quantitative contrast-enhanced MRA 31 (Supp. Fig. S5). QSM is feasible for applications in other body parts including the breast, extremity, and abdomen (liver and kidney) for studying hemorrhage, metabolic oxygen consumption, mineral distribution, and contrast agent kinetics 96.

### QSM of Tissue Complexity: Multiple Species and Microstructures

QSM techniques have started to proliferate, an indication that QSM is a fertile field for innovation. This review so far has focused on modeling a voxel of tissue with a scalar susceptibility. Here we briefly survey investigations to model MRI signal with tissue complexity: multiple species of different chemical shifts, subvoxel structures, and molecular structures.

### Nonlinear Phase Behavior of Multiple Spectral Species, Long TE, Large Voxel

The signal model in Eq. 5 may be regarded as a first order (linear temporal phase evolution) approximation, which may be good enough for many brain applications. For imaging other body parts, there may be signal contribution from proton sources other than free water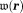, such as fat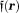 with chemical shift (characterized by a constant frequency offset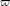 ∼ −3.4 ppm). Eq. [14] can be generalized to account for the chemical shift effects on signal phase 217,218. The spatial smoothness of the tissue magnetic field can be used for fat–water separation 219–221:

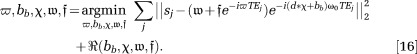
16

Note that the signal phase from a voxel is now nonlinear in its temporal evolution. Initial results in solving Eq. 16 are very encouraging, promising to extend QSM to body parts with fat (Fig. 14). The approximation in Eq. 5 works very well in most imaging situations but may break down in the presence of unusually strong susceptibility sources, long TEs, large voxels, or a combination of these factors. We may need to include higher-order terms in the evaluation of the exponential and include contributions from the neighboring voxels using an accurate voxel sensitivity function 44,222. These complications lead to a voxel signal phase that varies nonlinearly with TE.

### Signal Behavior with Subvoxel Structure

There is growing interest in modeling tissue microstructure using MRI 215,223–231. Subvoxel structures may be characterized as gradients and higher-order spatial derivatives in spin density and magnetic field 232. These violations of the smoothness assumption in digitizing Eq. 2 result in voxel signal phases with nonlinearly temporal evolutions. More useful models may include specific geometries for the underlying tissue microstructure such as solid or hollow cylinders for capillaries, fibers, and other linear microstructure (Supp. Fig. S6), also leading to phase nonlinear in time 225,227,233. Microstructures may be considered as static and observer water as undergoing rapid random motion. The ergodic hypothesis may be assumed: the sum over the observer water path becomes the sum over the ensemble distribution that is proportional to spin density. Then, voxel signal may be modeled as the sum of the contributions from water protons inside magnetic microstructures or compartments (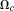) within the voxel. An example compartment is the cylinder or generalized Lorentz model 225. When water exchange among compartments is small, the signal model is a simple extension of Eq. 5,

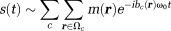
17

Here, for a given subvoxel compartment model, Maxwell’s Equations can be used to determine the field’s dependence on subvoxel structures (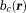) such as their orientations and underlying molecular susceptibility anisotropies 225,227,233. With a sufficient number of measurements, the compartmental susceptibility may be estimated from the MRI signals:

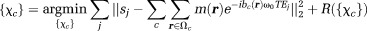
18

### Susceptibility Tensor

The diamagnetic susceptibilities of anisotropic molecules (Supp. Fig. S6) must be described by recognizing the susceptibility in Eq. 2 as a tensor. If all types of anisotropic molecules are sufficiently smoothly distributed in the space, and the spatial dispersion of phase accruals is sufficiently small in a voxel—as assumed in Eq. 5—then the corresponding digital form of Eq. 2 with tensor susceptibility can be used, forming the foundation for susceptibility tensor imaging (STI) 234. Group symmetry theory suggests that susceptibility anisotropy can only be observed in a voxel if and only if anisotropic molecules are arranged orderly on a macroscopic scale 235,236. The increased number of variables in STI requires acquisitions at many orientations 237, which may be reduced by using prior information obtained from diffusion tensor imaging 235,236. Similar to Eq. 17, subvoxel structures may be incorporated into the MRI signal equation, introducing phase nonlinear in time and other complexities 233. The most interesting biomaterial demonstrating susceptibility anisotropy may be myelin 107,238, and the assessment of myelin integrity using MRI remains an important unmet clinical need.

## CONCLUSION

Magnetic susceptibility directly reflects the molecular electron cloud behavior in the main magnetic field. Tissue susceptibility effects can be readily sensitized in MRI, for example using the widely available GRE sequence. Maxwell’s equations and the MRI signal equation can be used to quantitatively model the relationship between MRI signal and tissue susceptibility. Regularization is necessary to obtain a unique solution for determining the tissue susceptibility map from the acquired MRI signal, which is an ill-posed problem due to the lack of MRI signal in the background and the zeroes in the dipole kernel. The current status of QSM is very encouraging. The first order solution of QSM can be robustly obtained using physically meaningful regularizations, including the Bayesian approach. QSM has promising clinical and scientific applications that involve large susceptibility changes by hemoglobin, ferritin, calcification, and contrast agents. The investigations of higher order solutions have also been initiated, including studies of important magnetic anisotropies and tissue microstructures.
