# Supplementary material for: Quantitative susceptibility mapping (QSM): Decoding MRI data for a tissue magnetic biomarker
Source: Magn Reson Med. 2014 Jul 17;73(1):82–101. doi: 10.1002/mrm.25358 (PMC4297605; doi:10.1002/mrm.25358)
Supplement: Supplementary file 1 [file mrm0073-0082-sd1.docx]

**Supplemental Materials**

Here we provide a brief summary of 1) the molecular physics of tissue magnetism, 2) validation data and 3) validation results for QSM algorithms.

1. **TISSUE MAGNETISM – MOLECULAR AND PHYSICAL ORIGIN**

Tissue magnetic property is determined largely by the electronic configuration of its molecules. When all the magnets of the electron spins and orbits in a molecule exactly balance out to a zero net magnetic moment, bringing the molecule to the scanner $\boldsymbol{B}_{0}$ (increasing field) would induce currents in the molecule in such a direction as to oppose the field increase (Lenz’s law), or induce a magnetic moment in the molecule in the opposite direction of $\boldsymbol{B}_{0}$, which is called diagmanetism. When there is a net magnetic moment from the electron spins and orbits, the moment will experience a torque from $\boldsymbol{B}_{0}$ to line up with $\boldsymbol{B}_{0}$ in addition to the disorganizing force of thermal motion, resulting in a net magnetic moment parallel to $\boldsymbol{B}_{0}$, which is called paramagnetism. Let’s consider the example of hemoglobin, the iron-containing protein in red blood cells that transports oxygen ([1](#_ENREF_1)). Without oxygen (deoxyhemoglobin), hemoglobin’s iron ions (Fe^2+^) have 4 unpaired and 2 paired 3d electrons with a net magnetic moment of $2g_{e}\mu_{B}$ ($g_{e}=$2.002319 and $\mu_{B}=$9.274009×10^−24^ J·T^−1^), making deoxyhemoglobin strongly paramagnetic. When oxygen combines with deoxyhemoglobin, oxygen pulls Fe^2+^ into the porphyrin ring; this splits the Fe^2+^ 3d orbits, causing these 6 electrons to pair up in the 3 low energy levels with a zero net moment. Consequently, oxyhemoglobin is weakly diamagnetic ([2](#_ENREF_2),[3](#_ENREF_3)). During oxygen consumption, weakly diamagnetic oxyhemoglobin transforms into strongly paramagnetic deoxyhemoglobin, which forms the foundation of brain functional MRI (fMRI) ([4](#_ENREF_4)).

**Physics of Molecular Magnetic Polarizability**

This hemoglobin example suggests that quantum physics and chemistry are needed to understand tissue molecular magnetism ([2](#_ENREF_2),[5](#_ENREF_5)). In quantum physics, microscopic electrons have wave-particle duality. They are represented by wave functions characterized by energy, momentum, and angular momentum (with orbital angular momentum $\boldsymbol{l}\hbar$ and spin $\boldsymbol{s}\hbar$). An electron’s orbital angular momentum $\boldsymbol{l}\hbar$ generates a magnetic moment of $-\mu_{B}\boldsymbol{l}$ ([6](#_ENREF_6),[7](#_ENREF_7)), and its spin $\boldsymbol{s}\hbar$ generates a magnetic moment $-g_{e}\mu_{B}\boldsymbol{s}$ as explained by quantum electrodynamics ([8](#_ENREF_8)). Therefore, an electron’s total magnetic moment is

$\mathcal{m}_{e}=-\mu_{B}\left( \boldsymbol{l}+g_{e}\boldsymbol{s} \right)$.

[s1]

When an electron is brought into a constant magnetic field $\boldsymbol{B}_{0}$ along the z-axis, it gains a magnetic energy ([7](#_ENREF_7),[9](#_ENREF_9),[10](#_ENREF_10)) equal to $-\mathcal{m}_{e}\cdot\boldsymbol{B}_{0}\boldsymbol{=}\mu_{B}(\boldsymbol{l}+g_{e}\boldsymbol{s})\cdot\boldsymbol{B}_{0}$. Additionally, the electron at position $\boldsymbol{r}$ also gains a momentum $(-e\boldsymbol{B}_{0}\times\boldsymbol{r}/2)$ from the vector potential of $\boldsymbol{B}_{0}$ ([6](#_ENREF_6),[11](#_ENREF_11)), which has an energy $\frac{e^{2}}{8m_{e}}\left( \boldsymbol{B}_{0}\times\boldsymbol{r} \right)^{2}$. Summing over all electrons (with indices $j$) in a molecule, the total energy or the Hamiltonian $\mathcal{H}$ evaluated at a given state of the molecule is ([7](#_ENREF_7))

$$\mathcal{H=}\mathcal{H}_{0}+\mu_{B}\left( \sum_{j} \boldsymbol{l}_{j}+g_{e}\sum_{j} \boldsymbol{s}_{j} \right)\cdot\boldsymbol{B}_{0}+\frac{e^{2}}{8m_{e}}\sum_{j} \left( \boldsymbol{B}_{0}\times\boldsymbol{r}_{j} \right)^{2}.$$

[s2]

Here, the first term represents the electrostatic energy of the nuclei and electrons (including electron coupling ([7](#_ENREF_7))) that is independent of $\boldsymbol{B}_{0}$ and $\mathcal{H}_{0}\sim eV$; at $B_{0}=$3T the second term is ~ $\mu_{B}B_{0}=1.7\times{10}^{-4}eV$, and the third term is $\sim\frac{\left( \mu_{B}B_{0} \right)^{2}}{eV}\approx3\times{10}^{-8}eV$ ([12](#_ENREF_12)). Therefore, the $\boldsymbol{B}_{0}$ effect can be treated as a small perturbation on the molecular electron cloud. The nuclei also gain a $\boldsymbol{B}_{0}$-dependent energy as we know from nuclear magnetic resonance ([13](#_ENREF_13)), but the nuclear contribution is inversely proportional to the nuclear mass (~${10}^{3}m_{e}$) and is negligibly small compared to the electronic contribution. For a molecule at a given state (as characterized by its electron cloud) with energy $\mathcal{H}$ in a field $\boldsymbol{B}_{0}$, its magnetic moment $\mathcal{m}$ is ([5](#_ENREF_5),[14](#_ENREF_14))

$$\mathcal{m}=-\frac{\partial\mathcal{H}}{\partial\boldsymbol{B}_{0}}=-\mu_{B}\left( \sum_{j} \boldsymbol{l}_{j}+g_{e}\sum_{j} \boldsymbol{s}_{j} \right)-\frac{e^{2}}{4m_{e}}\sum_{j} (r_{j}^{2}-\boldsymbol{r}_{j}\boldsymbol{\bigotimes}\boldsymbol{r}_{j})\boldsymbol{B}_{0}.$$

[s3]

**Unpaired electrons generate paramagnetic moment**. The first term in Eq.s3 may be estimated in most situations in MRI with two approximations. The first approximation is the standard atomic electronic configuration as determined by Pauli’s exclusion principle ([6](#_ENREF_6)) and Hund’s rule of multiplicity ([2](#_ENREF_2)) for small molecules. Examples are the ferritin Fe^+3^ core with a half-filled 3d^5^ of $\sum_{j} \boldsymbol{l}_{j}=0,\sum_{j} \boldsymbol{s}_{j}\boldsymbol{=5/2}$ and the FDA-approved gadolinium contrast agents Gd^+3^ with a half-filled 4f ^7^ of $\sum_{j} \boldsymbol{l}_{j}=0,\sum_{j} \boldsymbol{s}_{j}\boldsymbol{=7/2}$. The second approximation is the angular momentum quench ($\sum_{j} \boldsymbol{l}_{j}=0$, then $\sum_{j} \boldsymbol{s}_{j}$ over unpaired electrons) for large molecules. An example is hemoglobin Fe^+2^ with $\sum_{j} \boldsymbol{s}_{j}\boldsymbol{=2}$ for deoxyhemoglobin with 4 unpaired 3d-electrons and $\sum_{j} \boldsymbol{s}_{j}\boldsymbol{=0}$ for oxyhemoglobin with no unpaired electrons ([2](#_ENREF_2)). For these biomolecule examples, the second energy term in Eq.2 is $g_{e}\mu_{B}B_{0}S_{z}, with S_{z}\in\left\{ -S, -S+1, \ldots, S-1, S \right\}, \boldsymbol{S}=\sum_{a} \boldsymbol{s}_{a}$ ([6](#_ENREF_6)). The ground state has the lowest energy $-Sg_{e}\mu_{B}B_{0}$ with all unpaired electrons’ magnetic moments parallel to $\boldsymbol{B}_{0}$ (paramagnetism). For tissue at body temperature ($\mathcal{T}=37℃$, $k_{B}\mathcal{T}=2.67\times{10}^{-2}eV$), the total spin can be in all its possible states according to Boltzmann’s distribution, resulting in the following paramagnetic magnetic moment ([5](#_ENREF_5)):

$$\mathcal{m}_{para}=\frac{\sum_{S_{z}=-S}^{S} \left( -g_{e}\mu_{B}S_{z} \right)exp(-\frac{S_{z}g_{e}\mu_{B}B_{0}}{k_{B}\mathcal{T}})}{\sum_{S_{z}=-S}^{S} exp(-\frac{S_{z}g_{e}\mu_{B}B_{0}}{k_{B}\mathcal{T}})}\cong\frac{g_{e}^{2}S\left( S+1 \right)\mu_{B}^{2}B_{0}}{3k_{B}\mathcal{T}}\equiv\beta_{para}B_{0}.$$

[s4]

Thermal motion spreads the possible states of the total spin almost equally over positive and negative $S_{z}$ values, resulting in only a small fraction ($\sim\mu_{B}B_{0}/k_{B}\mathcal{T=}6.4\times{10}^{-3}$) of “alignment” with $\boldsymbol{B}_{0}$ (Curie’s Law).

**Electron orbits generate diamagnetic moment**. The second term in Eq.s3 is always negative or diamagnetic (antiparallel to $\boldsymbol{B}_{0}$) for spherical electron clouds ([7](#_ENREF_7),[10](#_ENREF_10)): $-\frac{e^{2}B_{0}}{6m_{e}}<\sum_{j} r_{j}^{2}>$. In general, the second term in Eq.s3 depicts an induced magnetization with its direction related to $\boldsymbol{B}_{0}$ by a diamagnetic polarizability tensor with a negative trace:

[s5]

Here ${\hat{\boldsymbol{B}}}_{0}$ is a unit vector in the field $\boldsymbol{B}_{0}$ direction. Thermal motion hardly perturbs the electron cloud ($k_{B}\mathcal{T\ll}\mathcal{H}_{0}$), resulting in diamagnetism independent of temperature, unlike paramagnetism in Eq.s4 very sensitive to temperature. The electron clouds of anisotropic biomolecules (e.g., lipids) give rise to a nonzero diamagnetic polarizability tensor. Eqs.s4&s5 together define the molecular magnetic polarizability tensor . For a molecule with concentration $n$ in continuous space, the corresponding magnetization is.

**Magnetic Susceptibility of Biomaterials**

For a material being polarized to a magnetization $\mathfrak{m}$ by a magnetic field $\boldsymbol{b}$ (with the Lorentz correction as discussed in the main section), its magnetic susceptibility $\chi$ is defined as the coefficient between $\mathfrak{m}$ and the auxiliary H-field $(\boldsymbol{b/}\mu_{0}-\mathfrak{m)}$ ([10](#_ENREF_10),[14](#_ENREF_14)): $\mathfrak{m}=\chi(\boldsymbol{b/}\mu_{0}-\mathfrak{m})$. For all known biomaterials, $\chi\ll1, \boldsymbol{b\cong}\boldsymbol{B}_{0}$, and susceptibility as a tensor in general can be expressed as:

[s6]

Therefore, tissue magnetic susceptibility can be estimated from the electronic configurations of its molecules according to Eq.s6.

1. **Validation data**

**Numerical Simulation.** A 256 × 256 × 98 numerical brain phantom was created from an actual quantitative susceptibility map followed by manual segmentation of different gray matter regions and assigning constant susceptibilities in these regions. The simulated susceptibility values were 0.19, 0.09, 0.09, 0.07, 0.04, 0, and -0.05ppm for the globus pallidus, caudate nucleus, putamen, thalamus, cortical gray matter, cerebrospinal fluid, and white matter, respectively. The field perturbation of the model was calculated by fast forward field computation (B0 = 3 T in superior–inferior direction) and converted to GRE phase values at 11 TEs with the first TE at 2.6ms and an echo spacing of 2.6ms. The noise standard deviation was set as 0.02 arbitrary unit (a.u.) and signal intensities varied from 1 to 1.6a.u. in the parenchyma, so that the signal-to-noise ratio (SNR) for any voxel was at least 50. These values were empirically determined from in vivo human brain scans. The noise was simulated in real parts and imaginary parts independently.

**Phantom experiment.** A 2% agarose gel phantom containing five balloons of gadolinium solution (Magnevist, Berlex Labrotories, Wayne, NJ) was constructed. The highest concentration of the gadolinium was 0.5% followed by two-fold dilutions, leading to susceptibility values of 0.8, 0.4, 0.2, 0.1, and 0.05 ppm. This phantom was scanned on a 3T scanner (HDx, GE healthcare, Waukesha, WI) using a multi-echo gradient echo sequence with the following parameters: 8 TEs evenly spaced between 5 and 40 ms; TR=70 ms; acquisition matrix 130×130×86; voxel size =1×1×1 mm^3^; flip angle =15°; bandwidth =480Hz/pixel. The phantom was scanned from 12 different orientations with identical imaging parameters for the COSMOS calculation.

**In vivo experiment.** The human study was approved by our Institutional Review Board. One healthy volunteer was recruited to perform MR brain imaging on a 3.0-T scanner (HDx, GE Healthcare, Waukesha, WI, USA) with an eight-channel head coil. A 3D multi-echo spoiled gradient echo sequence was used. Imaging parameters were as follows: TEs = 5~50ms with an echo spacing of 5ms; TR = 55 ms; voxel size = 1 × 1 × 1 mm3; matrix size = 240 × 240 × 146; Bandwidth = 260Hz/pixel, flip angle (FA) = 15°, and parallel imaging with a reduction fraction of two. The same parameters were used to scan this subject five times for the COSMOS calculation, with the subject’s head positioned in neutral, chin down, chin up, left ear to shoulder and right ear to shoulder.

There are nuances in numerical optimization methods. To be faithful to the published methods, the following care was taken when implementing various methods: 1) the MATLAB codes were proofread by the original authors (TSVD, TKD and iSWIM), 2) the MATLAB codes from the original authors were used (MEDI and TVSB), 3) or the data were processed by the original authors and the results were returned (CSC and HEIDI). For $\boldsymbol{k}$-space approached (TSVD, TKD and iSWIM), the truncation threshold was set to 0.1 for all three experiments after consulting with the original authors. The regularization parameter (in front of the regularization term) was $3.3\times{10}^{-4}$, $3.3\times{10}^{-3}$, and $1.6\times{10}^{-3}$ in MEDI and $1.0\times{10}^{-3}$, $1\times{10}^{-2}$, and $6.3\times{10}^{-4}$ in TVSB for numerical simulation, phantom experiment, and in vivo experiments, respectively. The regularization used in CSC and HEIDI was determined by the original authors according to the optimized parameters from their respective papers. These regularization parameters are also summarized in Table S1.

1. **Validation results**

**Numerical Simulation.** All methods yielded satisfactory image qualities in the numerical simulation demonstrated that (Fig. S1a). Compared to the truth, there were minimal streaking on TSVD, TKD and iSWIM reconstructed images, while MEDI, CSC, HEIDI, and TVSB eliminated the streaking. The estimated susceptibility had good linear relationships with the truth (R^2^>0.8) in all cases (Fig. S1b). Among all the cases, the MEDI reconstructed QSM had the best accuracy (slope closest to 1). Closed-form solutions such as TSVD and TKD had the shortest calculation time (Table S2).

**Phantom experiment.** Substantial streaking artifacts were observed in k-space based methods that constrain solutions’ energies at the cone region, such as TSVD, and TKD (Fig. S2a). Although iterative filtering performed in iSWIM method reduced streaking (e.g. arrows in Fig. S2a), the most significant improvement was observed in Bayesian regularized dipole inversion such as in MEDI, CSC, HEIDI and TVSB. Strongest R2* values were observe at the interface between gadolinium balloons and the surrounding agarose gel. The calculated volume susceptibility can be further converted to gadolinium concentrations by dividing gadolinium’s molar susceptibility 308ppmL/mol. Quantitatively, Bayesian regularized methods had good linear relationships with the truth (R^2^>=0.8) (Fig. S1b), while most methods’ slopes deviated less than 10% from unity. Again, closed-form solutions such as TSVD and TKD had the shortest calculation time (Table S1). It was also noted that there was a faint halo surrounding the balloon with the highest gadolinium concentration in the COSMOS reconstruction (arrow). This was potentially due to misregistration when applying the linear image registration ([15](#_ENREF_15),[16](#_ENREF_16)) to register non-rigid balloons.

**In vivo experiment.** The in vivo brain MRI Fig. S3a demonstrated that all methods successfully generated QSMs with the major iron-laden structures clearly recognizable. Streaking artifacts not seen on COSMOS were observed in TSVD and TKD, and these variations appeared to originate from veins. iSWIM reduced but did not eliminate these artifacts. MEDI, CSC, and HEIDI yielded QSM images similar to COSMOS. Over-blurring was observed in TV. The strong contrasts between cortical gray and white matter seen on QSM images were almost invisible on the R2* map (arrow). In the linear regression analysis Fig. S3b, although MEDI had the highest slope and R^2^, the values were still far from satisfactory. Closed-form solutions such as TSVD and TKD had the shortest calculation time (Table S1).

Table S1. Regularization parameter

|  | Simulated brain | Gadolinium phantom | In vivo brain |
| --- | --- | --- | --- |
| MEDI^*^ | $3.3\times{10}^{-4}$ | $3.3\times{10}^{-3}$ | $1.6\times{10}^{-3}$ |
| TVSB^*^ | $1.0\times{10}^{-3}$ | $1\times{10}^{-2}$ | $6.3\times{10}^{-4}$ |
| TSVD^**^ | 0.1 | 0.1 | 0.1 |
| TKD^**^ | 0.1 | 0.1 | 0.1 |
| iSWIM^**^ | 0.1 | 0.1 | 0.1 |
| CSC^***^ |  |  |  |
| HEIDI^***^ |  |  |  |

* Regularization parameters are placed in front of the regularization term.

** Regularization parameters refer to the truncation level.

*** Unspecified regularization parameters. Criteria for choosing regularization parameters described in original papers.

Table S2. Calculation time

|  | Simulated brain | Gadolinium phantom | In vivo brain |
| --- | --- | --- | --- |
|  | Time (sec) | Time (sec) | Time (sec) |
| TSVD | 1.5 | 0.4 | 1.7 |
| TKD | 1.5 | 0.4 | 1.8 |
| iSWIM | 8.8 | 3.4 | 14 |
| MEDI | 195 | 173 | 1008 |
| CSC^(1)^ | 1074 | 2886 | 3463 |
| HEIDI^(2)^ | 512 | 227 | 715 |
| TVSB | 11 | 9.2 | 40 |

All the calculation were performed on a personal computer (PC) equipped with Intel® Core i7-3770k CPU @ 3.5GHz and 32 gigabytes (GB) of memory, except

(1) was a personal laptop with Intel Core i5-M2450 CPU @ 2.5GHz and 8GB of memory, and

(2) was a PC with Intel Core i5-2320 CPU @ 3.00 GHz and 16GB of memory.

FIG. S1a. Comparison of various QSM reconstruction methods on a numerical simulation

QSM images are reconstructed using various methods from left to right and then top to bottom: TSVD, TKD, iSWIM, MEDI, COSMOS, CSC, HEIDI, and TVSB.


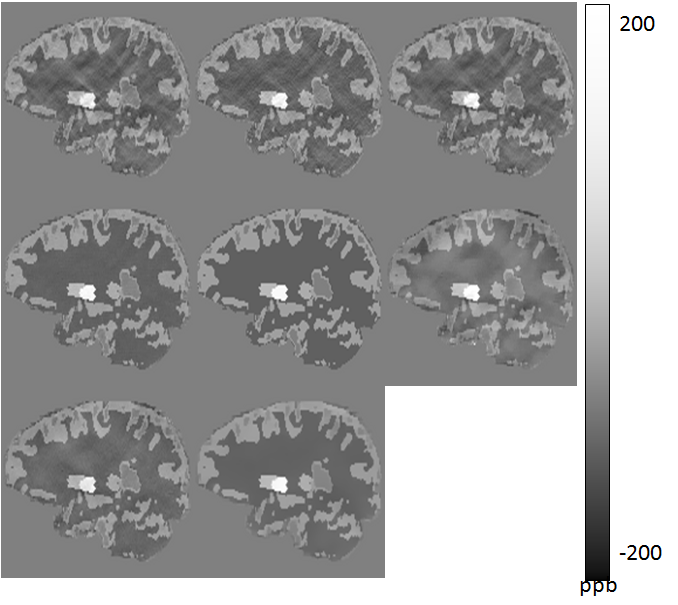


FIG. S1b. Linear regression between various QSM reconstruction methods and the truth on a numerical simulation.


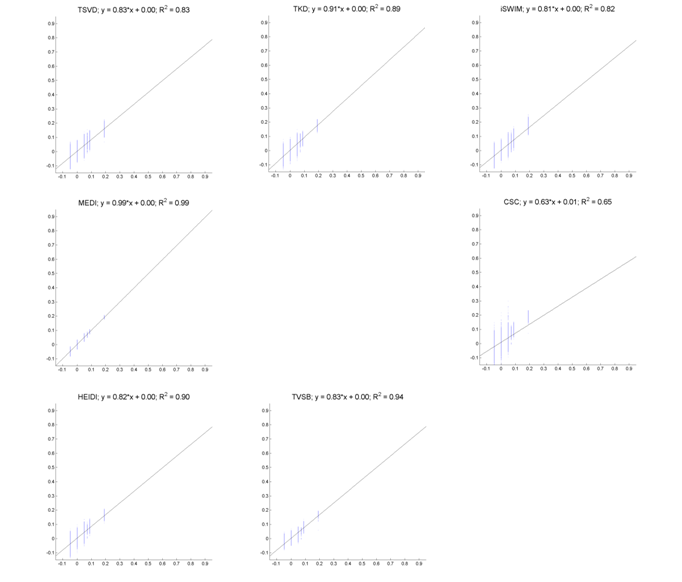


FIG. S2a. Comparison of various QSM reconstruction methods on a Gadolinium phantom

QSM images are reconstructed using various methods from left to right and then top to bottom: TSVD, TKD, iSWIM, MEDI, COSMOS, CSC, HEIDI, TVSB, and R2* map.


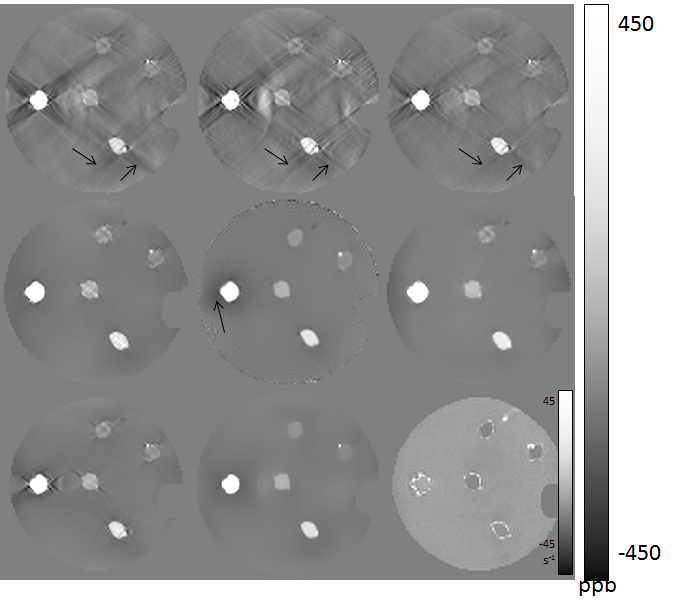


FIG. S2b. Region of interest based linear regression between various QSM reconstruction methods and the prepare concentrations on a gadolinium phantom.

FIG. S2c. Linear regression between various QSM reconstruction methods and COSMOS on a gadolinium phantom.


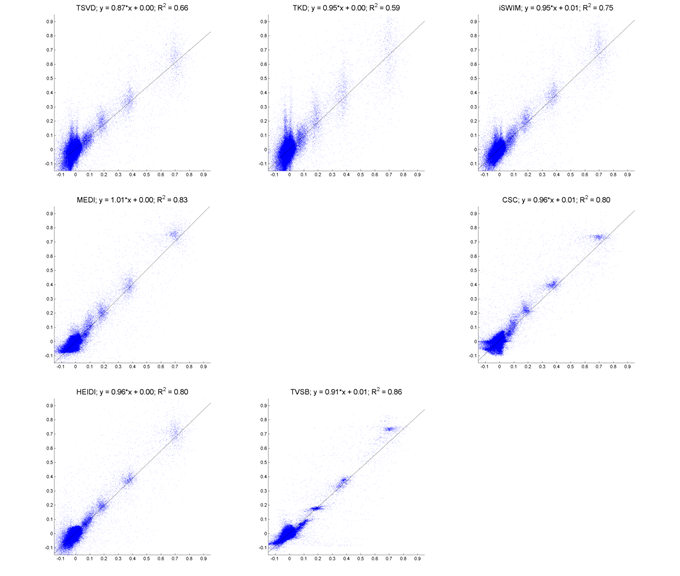


FIG. S3a. Comparison of various QSM reconstruction methods on a healthy volunteer

QSM images are reconstructed using various methods from left to right and then top to bottom: TSVD, TKD, iSWIM, MEDI, COSMOS, CSC, HEIDI, TVSB, and R2* map.


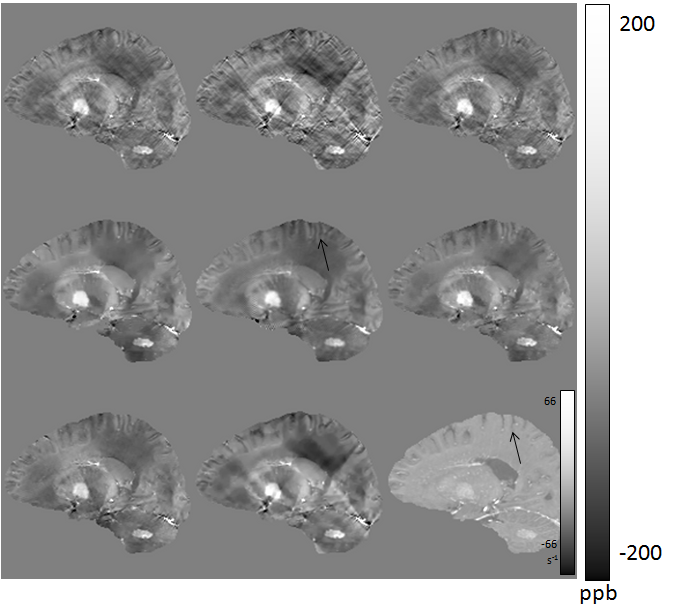


FIG. S3b. Linear regression between various QSM reconstruction methods and COSMOS on a healthy volunteer.


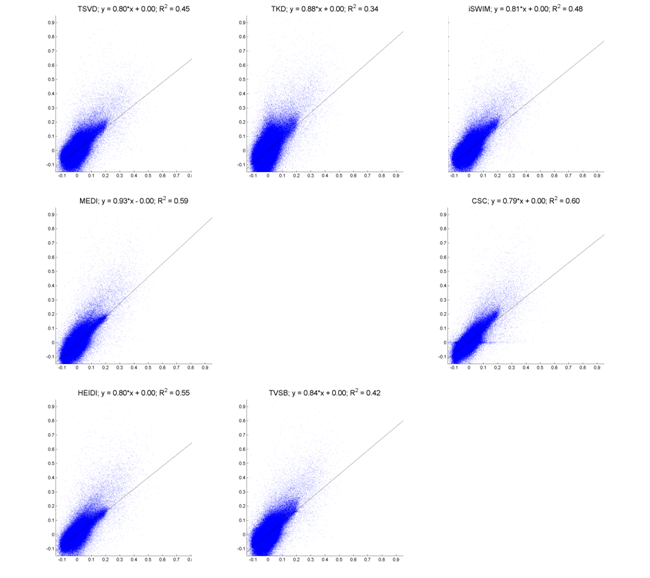


FIG. S4. EXPLANATION OF USING QSM TO QUANTIFY PARAMAGNETIC CONTRAST AGENTS.

(i) The inner sphere interaction for relaxation enhancement by a contrast agent (CA) molecule requires bound water in contact with CA. (ii) The magnetic field of the CA molecule extends far beyond the inner sphere and can be sensitized by phases of surrounding water proton spin.
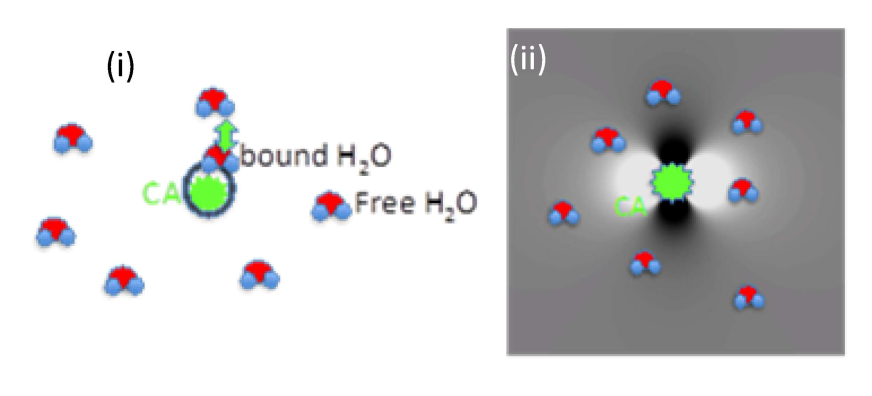


FIG. S5. QSM APPLICATIONS IN THE AORTA

Left image panel: In a dynamic Gd-enhanced aorta MRI, magnitude (1st row) and phase (2nd row) images every ~4.5sec are shown (numbers indicate time in sec) to demonstrate magnitude saturation at 14.8sec while the phase kept increasing until 19.4s. Right graph: QSM was applied to phase images at each time frame to generate the Gd concentration ([Gd]) time course of in the aorta (green, right), from which a cardiac output was estimated according to the indicator-dilution theory. The QSM-derived cardiac output agreed well with the phase-contrast flow measurement. The T1 enhancement of the magnitude was also used to generate a [Gd] time course in the aorta (blue, right), showing ~50% saturation at the peak moment.


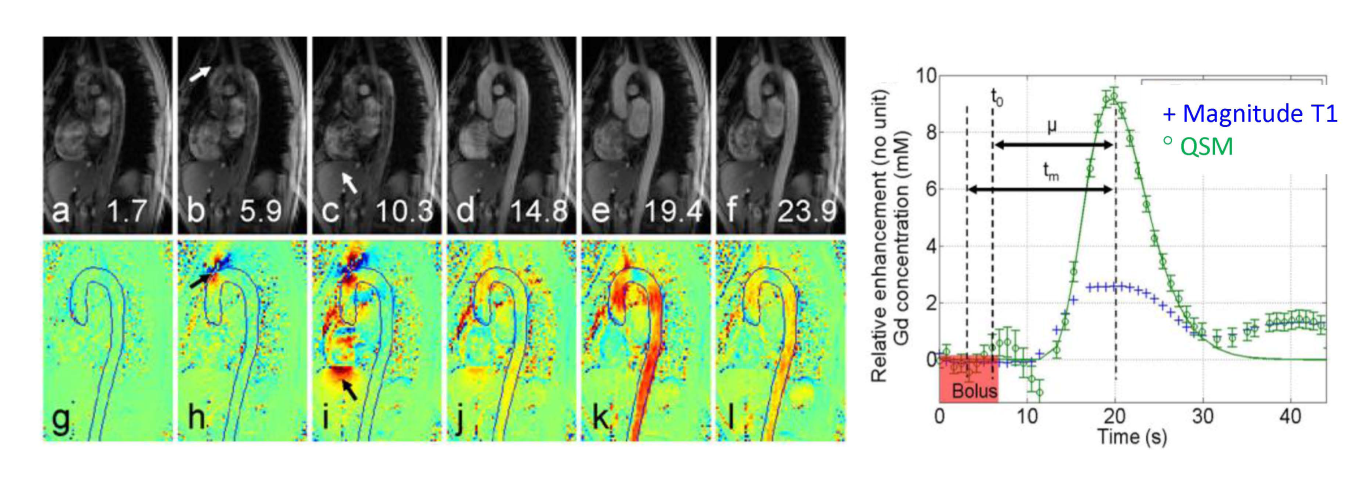


FIG.S6. SUBVOXEL STRUCTURE AND SUSCEPTIBILITY TENSOR.

(i) Solid cylinder model for subvoxel structure. (ii) Cylindrical shell model for subvoxel structure. (iii) Cylindrically symmetric susceptibility tensor (CSST) model for anisotropic molecules such as lipids. The tensor frame is defined by the axes along and , which is with respect to $\boldsymbol{B}_{0}$. (iv) Molecular organization of lipids in a cross-section of the myelin: lipids represented by ellipsoids with their long-axes in the cross-section.


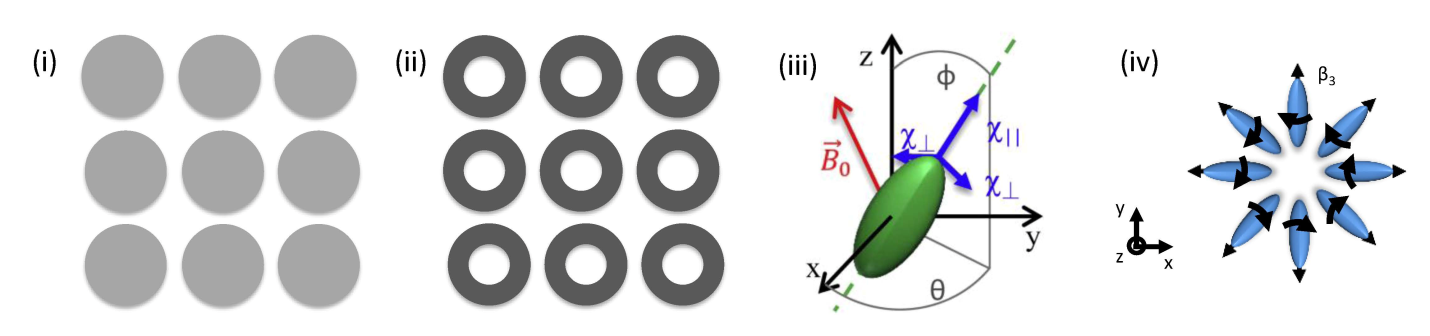


**References for supplemental materials:**

1. Perutz MF. Stereochemistry of cooperative effects in haemoglobin. Nature 1970;228(5273):726-739.

2. Pauling L. General Chemistry: Dover Publications; 1988.

3. Pauling L, Coryell CD. The Magnetic Properties and Structure of Hemoglobin, Oxyhemoglobin and Carbonmonoxyhemoglobin. Proc Natl Acad Sci U S A 1936;22(4):210-216.

4. Ogawa S, Lee TM, Kay AR, Tank DW. Brain magnetic resonance imaging with contrast dependent on blood oxygenation. Proc Natl Acad Sci U S A 1990;87(24):9868-9872.

5. Kahn O. Molecular magnetism: VCH; 1993.

6. Feynman RP, Leighton RB, Sands M. Lectures on physics, volume III: Addison-Wesley publishing company; 1965.

7. Lifshitz EM, Landau LD. Quantum mechanics, non-relativistic theory. Oxford: Butterworth-Heinemann; 1981.

8. Schwinger J. On quantum-electrodynamics and the magnetic moment of the electron. Phys Rev 1948;73(4):416-417.

9. Jackson JD. Classical electrodynamics, third edition: John Wiley and Sons, inc.; 1999.

10. Feynman RP, Leighton RB, Sands M. Lectures on physics, volume II: Addison-Wesley publishing company; 1965.

11. Maxwell JC. A treatise on electricity and magnetism. New York: Dover Publcations; 1954.

12. Wang Y. Principles of Magnetic Resonance Imaging: physics concepts, pulse sequences & biomedical applications: [www.createspace.com/4001776;](http://www.createspace.com/4001776;) 2012.

13. Abragam A. Principles of Nuclear Magnetism. International Series of Monographs on Physics 32: Oxford Science Publications; 1961. p 177.

14. Landau LD, Lifshitz EM, Pitaevskii LP. Electrodynamics of continuous media. Oxford: Butterworth-Heinemann; 1984.

15. Jenkinson M, Smith S. A global optimisation method for robust affine registration of brain images. Med Image Anal 2001;5(2):143-156.

16. Jenkinson M, Bannister P, Brady M, Smith S. Improved optimization for the robust and accurate linear registration and motion correction of brain images. Neuroimage 2002;17(2):825-841.
